# An Exception to Mental Simulation: No Evidence for Embodied Odor Language

**DOI:** 10.1111/cogs.12593

**Published:** 2018-02-14

**Authors:** Laura J. Speed, Asifa Majid

**Affiliations:** ^1^ Centre for Language Studies Radboud University; ^2^ Max Planck Institute for Psycholinguistics; ^3^ Donders Institute for Brain Cognition and Behaviour Radboud University

**Keywords:** Mental simulation, Embodiment, Memory, Olfaction, Audition

## Abstract

Do we mentally simulate olfactory information? We investigated mental simulation of odors and sounds in two experiments. Participants retained a word while they smelled an odor or heard a sound, then rated odor/sound intensity and recalled the word. Later odor/sound recognition was also tested, and pleasantness and familiarity judgments were collected. Word recall was slower when the sound and sound‐word mismatched (e.g., bee sound with the word *typhoon*). Sound recognition was higher when sounds were paired with a match or near‐match word (e.g., bee sound with *bee* or *buzzer*). This indicates sound‐words are mentally simulated. However, using the same paradigm no memory effects were observed for odor. Instead it appears odor‐words only affect lexical‐semantic representations, demonstrated by higher ratings of odor intensity and pleasantness when an odor was paired with a match or near‐match word (e.g., peach odor with *peach* or *mango*). These results suggest fundamental differences in how odor and sound‐words are represented.

## 1 Introduction

To‐day I think
Only with scents, — scents dead leaves yield,
And bracken, and wild carrot's seed,
And the square mustard field
“Digging” by Edward Thomas

How do we understand such rich descriptions of our perceptual world which evoke what we see, hear, feel, and smell? Embodied approaches to language comprehension propose this is done by mentally simulating through the brain's perception and action systems (e.g., Barsalou, 1999). That is, understanding word meanings recruits processes used to perceive the real‐world referent of a word. Mental simulation is considered different to mental imagery, which is explicit (Willems, Toni, Hagoort, & Casasanto, 2010). Instead, mental simulation should occur automatically and implicitly. This approach is in line with Wilson's (2002) fourth type of embodiment—that is, offline cognition is “body based.” Most evidence for such simulation, however, has focused on the dominant perceptual modality of vision (e.g., Kaschak et al., 2005; Martin, Haxby, Lalonde, Wiggs, & Ungerleider, 1995; Meteyard, Bahrami, & Vigliocco, 2007; Meteyard, Zokaei, Bahrami, & Vigliocco, 2008; Pulvermuller & Hauk, 2006), with the “lower senses,” such as smell, neglected. To understand the evocative olfactory experience invoked by Edward Thomas’s words above, do we also mentally simulate? By focusing primarily on vision, theories of embodiment are circumscribed in how much of language comprehension they explain (e.g., Speed, 2016), as are theories of psycholinguistics more generally (Levinson & Majid, 2014).

One study suggests mental simulation of odor during language comprehension does occur: González et al. (2006) found reading words with strong olfactory associations (e.g., *cinnamon*) activated the piriform cortex (the primary olfactory cortex). Yet behavioral evidence shows people often inaccurately name odors (e.g., Cain, 1979), at least in the West (e.g., De Valk, Wnuk, Huisman, & Majid, 2017; Majid, 2015; Majid & Burenhult, 2014; Wnuk & Majid, 2014). Imagining odors is similarly difficult (Crowder & Schab, 1995), and olfactory dreams are uncommon (Stevenson & Case, 2004), suggesting activation of olfactory representations without real odors is uncommon. In fact, Olofsson and Gottfried (2015) propose that there is a weak link between olfaction and language. In their neurocognitive model, olfaction and language are too “directly” connected: Olfactory and linguistic information is integrated at only the third synapse from olfactory receptors (Olofsson & Gottfried, 2015). This means that olfactory information receives little processing, leaving a coarse, underspecified representation at the point of lexical integration, according to the authors. From coarse representations, the model predicts that broader lexical categories are more likely to be activated, such as *fruit*, rather than more specific terms, such as *lemon*.

Mental simulation of odor then seems at odds with the proposed weak link between odor and language (Olofsson & Gottfried, 2015). For example, the results of González et al. (2006) imply that simply viewing words with olfactory associations can activate the olfactory system. We see three possible explanations for the discrepancies between the two proposals. First, the link between olfaction and language could be asymmetric. Olfactory language could accurately link to odor representations, but odor representations fail to activate odor names. In line with this, difficulties in identifying odors are alleviated when a list of odor names is available (De Wijk & Cain, 1994). The following two explanations relate to the granularity of odor activation. The activations to odor‐related words in piriform cortex might mirror the broad, coarse activations thought to be sent to language regions (cf., Olofsson & Gottfried, 2015). That is, an odor‐related word such as *cinnamon* may activate a similar region of the olfactory cortex to an odor‐related word such as *nutmeg*, because they have similar broad, or category, representations (e.g., spices). In fact, odor‐related words may activate the piriform cortex in an even coarser manner. There may be little difference in activation to an odor‐related word such as *shrimp* and an odor‐related word such as *rose*, which come from very different categories, if only the attribute of “odor” is simulated. If this were the case, then Edward Thomas's attempt at conjuring odors to mind in the quote above may be futile. This coarseness would contrast with mental simulation in the motor cortex, which is shown to be somatotopic (Hauk, Johnsrude, & Pulvermüller, 2004). A final possibility is that González et al.'s (2006) piriform activation was not caused by mental simulation of odor at all, but instead by mental processes unrelated to simulation, such as sniffing (Arshamian, personal communication). Simply sniffing odorless air can activate the piriform cortex (Sobel et al., 1998), and if participants sniffed in response to odor‐words, this could have led to piriform activation, especially since odor and control words were presented in separate blocks.

There is other evidence, however, that the olfactory cortex can be activated in an odor‐specific manner without olfactory stimulation. Zelano, Mohanty, and Gottfried (2011) found evidence of pre‐stimulus odor‐specific ensemble patterns in piriform cortex before the presentation of an odor in a target detection task. That is, when expecting a specific odorant, a feature‐specific odor template was generated. Regions of the brain involved in odor perception have also been found to be activated during mental imagery of odors (Djordjevic, Zatorre, Petrides, Boyle, & Jones‐Gotman, 2005; but see Palmiero et al., 2009), and when holding odors in working memory (Zelano, Montag, Khan, & Sobel, 2009). There is also evidence that people can experience specific and vivid olfactory hallucinations (Greenberg, 1995). This would suggest odor‐specific mental simulation is possible. Importantly, beyond explicit mental imagery, whether language has access to such representations is still an open question.

The present work therefore sets out to test whether mental simulation of olfactory information occurs during language comprehension and, if so, to assess the granularity of such simulation. To do this, we paired words with strong olfactory associations to real odors, and then assessed the effect of this pairing behaviorally. Combining linguistic stimuli with sensorimotor stimuli is a standard method to test for shared processing regions between language and perception/action. For example, lexical decisions to words describing upwards or downwards motion are hindered if presented with concurrent visual motion of matching direction (Meteyard et al., 2008). Conversely, detection of directional visual motion at threshold is hindered when passively listening to motion verbs of congruent direction (Meteyard et al., 2007).

One problem with adopting such a methodology with olfactory stimuli is that it is difficult to control the timing of odor presentation without an olfactometer; and even then neural processing of olfactory signals is in the order of around 200 msec, but only around 50 msec for vision and 10 msec for audition (Keetels & Vroomen, 2012; Khan & Sobel, 2004). Aside from differences in reaction time, another relevant dependent variable is memory performance. Memory effects have been found when combining actions with words. In the well‐studied “enactment effect,” or “subject‐performed task effect,” action phrases that are heard/read and then performed are remembered better than action phrases that are simply heard/read (e.g., Engelkamp, 2001). For example, participants recalled more surprising words than neutral words with a surprised face than a neutral face, and vice versa (Parzuchowski & Szymkow‐Sudziarska, 2008).

If odor‐related words and real odors activate overlapping regions of the brain, and both are active at the same time, this could make memory performance worse in one domain, because of competition for resources (as in Crawford, Cohn, & Kim, 2014). Consistent with this, Andrade and Donaldson (2007) found reduced odor recognition memory with a concurrent odor task during encoding compared to a concurrent verbal or visual task. Alternatively, memory performance could be improved because the overlap may increase overall activation, thereby strengthening the memory trace (as in Engelkamp, 2001; Palma, Garrido, & Semin, 2011; Parzuchowski & Szymkow‐Sudziarska, 2008).

This study combined words with strong olfactory associations and real odors, to test whether their combination affects memory for odors. According to the results of González et al. (2006), reading odor‐words should activate primary olfactory cortex. If so, this activation should affect subsequent processing of incoming odors, either through interference and hence competition, or facilitation due to increased activation. Interference should lead to poorer odor recognition, whereas facilitation should lead to better recognition. If, on the other hand, comprehending odor‐words does not recruit odor processing systems, then there should be no effect of words on odor memory.

Before investigating olfactory language, we first investigated the same research question with auditory language, to ensure that our experimental paradigm was appropriate. Previous research has shown that auditory information is simulated during language comprehension (e.g., Kiefer, Sim, Herrnberger, Grothe, & Hoenig, 2008), including such specific details as direction (Kaschak, Zwaan, Aveyard, & Yaxley, 2006) and distance (Winter & Bergen, 2012). We, therefore, expected to find effects of auditory simulation with the present paradigm, replicating previous findings and serving as an experimental paradigm check. We focused on audition rather than vision for two reasons: first, visual memory is extremely accurate (Cohen, Horowitz, & Wolfe, 2009), and could therefore result in ceiling effects. Second, using both pictures and written words would add an additional variable of within modality (visual pictures and words) versus between modality (odors and words), which would create a needless confound between modalities.

### 1.1 Current investigation

In Experiment 1, we assessed the validity of our experimental paradigm by testing the mental simulation of auditory information. We then tested our primary research question in Experiment 2; namely, do we mentally simulate olfactory information during language comprehension?

In Experiment 1, participants were given a word to remember followed by a sound clip which they rated on intensity. Then participants had to recall the original word they were given before the rating task. The word was either (i) the label of the sound they heard (e.g., the word *telephone* followed by the sound of a telephone: match condition); (ii) the label of another source object that sounded similar (e.g., word *alarm*—sound telephone: near‐match condition); (iii) the label of another source object with strong auditory associations (e.g., word *crickets—*sound telephone: mismatch condition); (iv) or a word with no auditory associations (e.g., word *marble*—sound telephone: neutral ^1^ condition). Finally, recognition memory for sounds was tested. Based on previous work (e.g., Kaschak et al., 2006; Kiefer et al., 2008; Winter & Bergen, 2012), we expected sound‐words would affect both sound perception and recognition. Since it has been shown that auditory simulations include details such as direction (Kaschak et al., 2006) and distance (Winter & Bergen, 2012), it seems auditory simulations may be fairly fine‐grained, and so we expected there to be a difference in sound recognition between the matching versus near‐matching and mismatching sound conditions.

Using the same paradigm, in Experiment 2 participants smelled a number of odors and odor recognition was tested later. Before smelling each odor, participants were given a word to remember. They then sniffed the odor and rated it on intensity, and thereafter recalled the original word. The word was either (i) the label of the odor they smelled (e.g., the word *garlic* followed by the smell garlic: match); (ii) the label of another source object that smelled similar, that is, had a similar smell quality (e.g., word *onion*—smell garlic: near‐match); (iii) the label of another source object with strong olfactory associations (e.g., word *soap*—smell garlic: mismatch); (iv) or a word with no (or low) olfactory associations (e.g., word *water*—smell garlic: neutral).

Retaining a word in memory while smelling an odor meant the word's meaning remained active during processing of the odor, and so provided a crucial test of what type of odor representation (if any) the words elicited. If mental simulation of odor does occur, we can then test for the specificity of olfactory representation based on the three types of odor‐words (match, near‐match and mismatch) we include, as well as the control condition (neutral). If words activate the olfactory cortex in a specific way, that is, specific to the particular odor, then only words that match the odor should affect the olfactory percept and later recognition of that odor. If, on the other hand, words activate the olfactory cortex at a coarse level, then both match and near‐match words for odors should affect perception and later recognition. A third possibility is that words with strong olfactory associations activate the olfactory cortex only in a very general manner. If so, then holding such words in memory should affect olfactory perception generally, affecting recognition of all odors, compared to neutral words. Finally, if the olfactory cortex is not involved in comprehension of words denoting objects with strong olfactory associations at all, there should be no difference between any of the conditions with odor‐words and the neutral condition. It could also be expected that the odors, in fact, affect recall of the *word*: smelling an odor may interfere with retention of the word if they are related in one of the manners described above (i.e., specific, coarse, or general). We therefore analyze word recall accuracy and response time, as well as odor recognition.

In addition to examining memory for words and sounds/odors in Experiments 1 and 2, we also examined how the representation of sound and odor stimuli was affected across conditions. Language influences perceived odor pleasantness, intensity, and familiarity (Ayabe‐Kanamura, Kikuchi, & Saito, 1997; Distel & Hudson, 2001; Herz, 2003; Rabin & Cain, 1984). These properties are enhanced when odors can be identified or when their name is available. Therefore, in addition to intensity, we also included two ratings as dependent variables: ratings of pleasantness and familiarity. On the basis of previous research, we predicted that odors would be rated as more pleasant, intense, and familiar if they were paired with a matching word. Moreover, if odors are represented in a coarse manner during simulation, we would expect the same enhancement in the near‐matching condition compared to the mismatching and neutral conditions; and if the simulations are very general indeed, then odor ratings should be higher in all three conditions with an odor‐related word compared to the neutral condition. It is unclear whether ratings of sound pleasantness, intensity, and familiarity would similarly be affected by language. It is possible that odor is more influenced by language than the other senses because odors are poorly located in space and difficult to identify (Herz, 2000).

To summarize, the following experiments set out to investigate the mental simulation of sound and odor, using a memory paradigm in which real sounds and odors were perceived while holding words in mind that reflected different categories of odors or sounds. If odor and sound‐words activate mental simulations of the respective sensory domain, we expect words will affect recognition for perceptual stimuli, and conversely, perceiving odors and sounds may affect recall of sensory words. Furthermore, we predict that odor‐words specifically will affect ratings of pleasantness, intensity, and familiarity of odors. Before the experiments are described in detail, we first describe the development of the linguistic stimuli for each experiment.

### 1.2 Sound and odor‐word norming

To select stimuli that appropriately reflected sound or odor information, we first conducted modality ratings of a number of nouns, choosing those that were judged to reflect odor or sound information.^2^ Following this, we conducted similarity ratings of pairs of words in order to create the “near‐match” and “mismatch” pairs.

### 1.3 Modality ratings

#### 1.3.1 Participants

Thirty‐three participants (7 males, average age 23.5, *SD* = 3.5) signed up for the ratings task through the Radboud University Sona system and were sent a link to complete a Qualtrics survey. Participants were paid for their time with shopping vouchers.

#### 1.3.2 Stimuli

Initially, lists of 100 words for each modality (visual, auditory, haptic, gustatory, olfactory) were created. We aimed to find an equal number of words with strong perceptual associations for each modality. To do this, we consulted the stimuli list of Lynott and Connell (2013), as well as Mulatti, Treccani, and Job (2014) for words with sounds associations, Barros‐Loscertales et al. (2012) for words with gustatory associations, and González et al. (2006) for words with olfactory associations. Despite our best attempts to curate equal numbers of words for each modality, some words overlapped between the olfactory and gustatory modality, leading to a list of 485 words in total. Three separate lists were randomly created to be rated by participants using a Qualtrics survey (Qualtrics, Provo, UT).

#### 1.3.3 Procedure

Participants were presented with each word separately and asked to rate to what extent the meaning of that word could be experienced by feeling through touch, hearing, seeing, tasting, and smelling, on a visual scale of 0 (not at all) to 5 (greatly) (following Lynott & Connell, 2009, 2013). For each modality, participants had to click on their chosen value to make their response. Participants were instructed to leave a word unrated if they were unsure of the meaning of the word.

### 1.4 Results

Words with an average auditory rating of 3 or more were considered “sound‐words” (68 words) and words with an average olfactory rating of 3 or more were considered “odor‐words” (73 words). From each list of words near‐match (e.g., *machine gun‐drill, beer‐cider*) and mismatch (e.g., *rocket‐bee, pineapple‐olive*) pairs were created based on the two experimenters’ intuition about similarity in smell and sound (69 near‐match and 69 mismatch sound pairs and 71 near‐match and 71 mismatch odor pairs). The pairs were then entered into a second Qualtrics survey to collect similarity ratings to check this intuition.

### 1.5 Similarity ratings

#### 1.5.1 Participants

Seventeen participants (3 males, average age 21.1, *SD* = 2.1) signed up to rate the word pairs through the Radboud University Sona system and were sent a link to complete the Qualtrics survey. Participants were paid for their time with shopping vouchers.

#### 1.5.2 Procedure

Participants were presented with word pairs and asked to imagine the sound or smell of the object denoted by the word and rate the similarity in sound or smell on a scale of 0 (not at all similar) to 7 (very similar). Sound and odor judgments were completed in two separate blocks with the order counterbalanced across participants. Participants were also asked to indicate if they did not know the meaning of any words. Words labeled unknown by three or more participants were not considered in the final item selection (eight sound‐words and five odor‐words).

### 1.6 Results

Similarity ratings were median split so that word pairs in the upper half of the data were considered near‐match and those in the lower half were considered mismatch. Words were then selected as targets based on their paired near‐match and mismatch (16 for sound 16 for odor). A word could only appear once in the experimental set either as the target, or as near‐match or mismatch. Due to a number of items being removed after participants had labeled them unknown, several targets did not have ratings for pairings with mismatches. We therefore had to choose additional words that did not have ratings, but that to us were clear matches (highlighted in italics in Tables 1 and 2), which was further confirmed by data from another set of participants. For similarity ratings, near‐match pairs were rated as significantly more similar than mismatch pairs for both sounds *t*(11) = 11.25, *p *< .001 and odors *t*(12) = 9.72, *p* < .001. A separate set of participants (*n* = 17) rated the additional pairs (in italics). Including all ratings, near‐match, pairs were rated as significantly more similar than mismatch pairs for both sounds *t*(15) = 10.24, *p *< .001 and odors *t*(15) = 11.85, *p* < .001. Using the Spoken Dutch corpus, words were matched in terms of frequency across target, near‐match and mismatch for both sounds *F*(2, 30) = 1, *p* = .38 and odors *F*(2, 30) = 1.38, *p* = .27 .

**Table 1:** Target sounds and near‐ and mismatch pairings

Target	Near Match	Similarity	Mismatch	Similarity
zee (sea)	golven (waves)	6.06	auto (car)	0.94
bom (bomb)	vuurwerk (firework)	5.65	saxofoon (saxophone)	0.59
telefoon (phone)	alarm (alarm)	3.29	krekels (crickets)	1.29
*regen (rain)*	*hagel (hail)*	*5.82*	*bel (bell)*	*1.59*
handdroger (hand dryer)	stofzuiger (vacuum cleaner)	4.76	video (video)	0.76
wolf (wolf)	hond (dog)	4.65	kuch (cough)	0.81
bij (bee)	zoemer (buzzer)	4.35	tyfoon (typhoon)	1.06
blender (blender)	boor (drill)	4.24	keyboard (keyboard)	0.71
ventilator (fan)	droger (dryer)	4.12	film (film)	1.18
machinegeweer (machine gun)	helikopter (helicopter)	4.06	piano (piano)	0.88
vogelzang (bird song)	fluitje (whistle)	3.94	grasmaaier (lawn mower)	0.59
applaus (applause)	trommel (drum)	3.35	olifant (elephant)	1.82
*harp (harp)*	*gitaar (guitar)*	*4.88*	*nies (sneeze)*	*1.59*
*trein (train)*	*raket (rocket)*	*3.29*	*sirene (siren)*	*3.12*
*triangel (triangle)*	*deurbel (doorbell)*	*5.06*	*waterval (waterfall)*	*2.53*
hakken (heels)	hoeven (hooves)	4.00	didgeridoo (digeridoo)	0.94

**Table 2:** Target odors and near‐ and mismatch pairings

Target		Similarity		Similarity
	Near Match		Mismatch	
*whisky (whisky)*	*rum (rum)*	*5*	*popcorn (popcorn)*	*1.88*
afwasmiddel (detergent)	zeep (soap)	4.88	azijn (vinegar)	0.65
verf (paint)	benzine (gasoline)	4.18	openhaard (fireplace)	0.71
perzik (peach)	mango (mango)	3.71	oregano (oregano)	0.65
parmezaan (parmesan)	brie (brie)	3.71	kattenvoer (cat food)	1.18
*nootmuskaat (nutmeg)*	*kruidnagel (cloves)*	*4.94*	*olijf (olive)*	*2.53*
sinaasappel (orange)	limonade (lemonade)	3.47	menthol (menthol)	0.41
peterselie (parsley)	basilicum (basil)	3.35	aardbei (strawberry)	0.35
cacao (cocoa)	koffie (coffee)	3.18	zalm (salmon)	0.24
karamel (caramel)	vanille (vanilla)	3.12	ananas (pineapple)	0.94
*rozemarijn (rosemary)*	*lavendel (lavender)*	*4.12*	*tequila (tequila)*	*2*
knoflook (garlic)	ui (onion)	2.71	citroengras (lemongrass)	0.65
zoethout (liquorice)	anijs (aniseed)	2.53	haarlak (hairspray)	1.29
cider (cider)	bier (beer)	2.59	banaan (banana)	1.06
koriander (coriander)	curry (curry)	2.12	sigaret (cigarette)	0.59
jasmijn (jasmin)	tijm (thyme)	2.12	rook (smoke)	0.76

Word pairs were placed into four experimental lists so that each word occurred in either the match, near‐match, mismatch, or a neutral condition (as baseline) across the experiment. This meant that there were four items per condition per list (Tables 1 and 2). In each experimental list there were four items in the neutral condition (same in each list). Neutral sound‐words had an average rating of >1.6 for the sound modality: *marmer* (marble), *verrekijker* (binoculars), *ladder* (ladder), *kiezel* (pebble); and neutral odor‐words had an average rating of 0 for the odor modality: *glitter* (glitter), *maan* (moon), *richel* (ledge), *scheermes* (razor).

## 2 Experiment 1: Sound

### 2.1 Method

#### 2.1.1 Participants

Sixty‐one new participants were recruited from the Radboud University Sona system (46 females, mean age = 23.41, *SD *= 5.41) and paid for their participation in shopping vouchers.

#### 2.1.2 Stimuli

Sixteen target sound‐words with a near‐match, mismatch, and neutral word were selected, as described in the norming section; and for the 16 target words, 16 sound clips were created. In addition, 16 distractor sounds were chosen for the sound recognition task which reflected sound‐words chosen from the norming procedure that had not been used in the experiment. The sounds used were taken from an online sound database freesound.org and were all real‐world sounds. Each sound clip lasted 4 seconds.

In addition, the Clarity of Auditory Imagery Scale (CAIS; Willander & Baraldi, 2010) was administered to participants as an intervening task between sound encoding and sound recognition. The CAIS includes 16 items for which the participant must imagine the sound of, for example, a clock ticking, a mobile phone ringing, and so on. This allowed us to later examine the relationship between auditory imagery and auditory memory. A better ability to imagine sounds may aid retention of sounds and later sound recognition.

#### 2.1.3 Design

There was one within‐participant factor with four conditions: match (sound and same sound‐word), near‐match (sound‐word for a similar sound), mismatch (sound and different sound‐word), and neutral (sound and non‐sound‐word).

#### 2.1.4 Procedure

**Figure 1:**
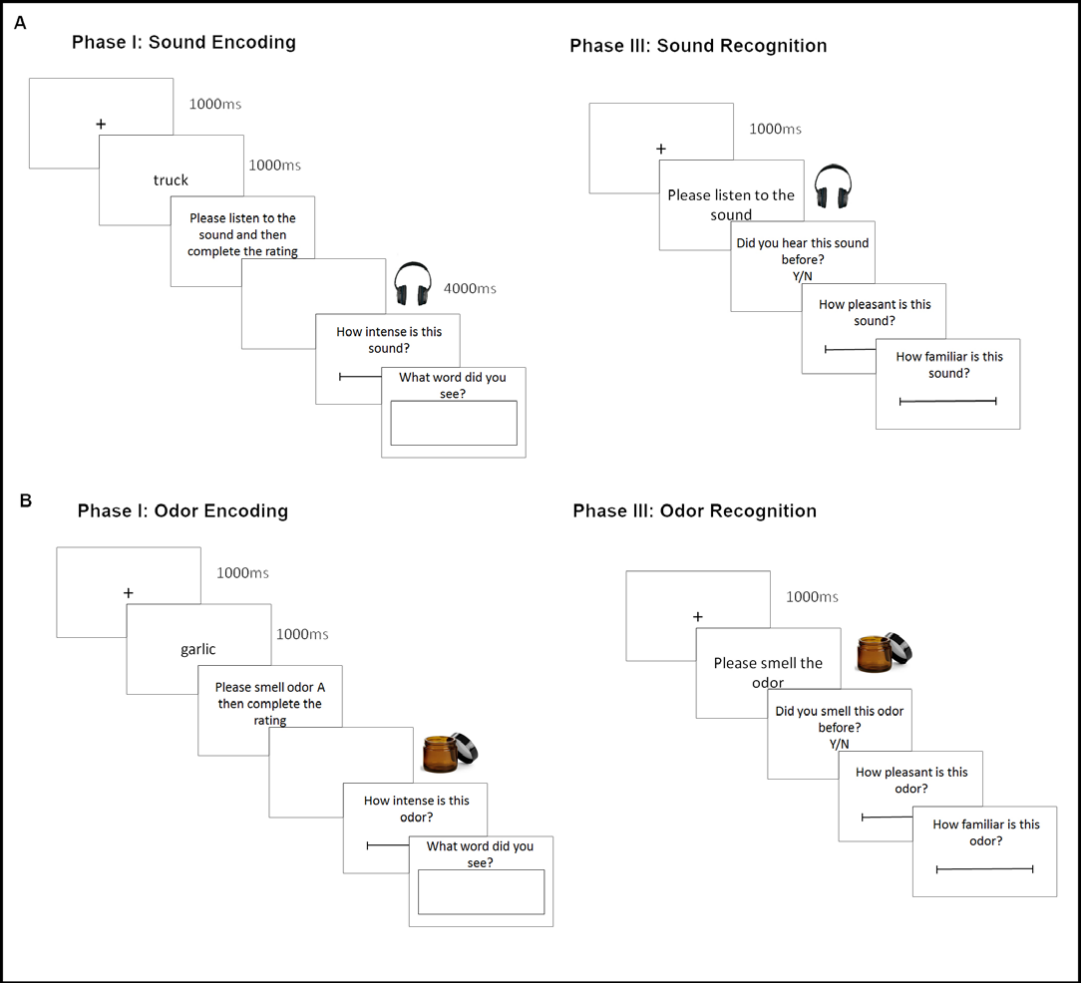
Encoding and recognition procedure for (A) Experiment 1 and (B) Experiment 2.

Participants were told that the experiment investigated multitasking, and they were informed of the procedure before providing informed consent. They were therefore aware that their memory for sounds would be tested. The experiment had three phases (see Fig. 1A).

Phase I—A trial proceeded as follows: Participants were first shown a word to be remembered; they then listened to a sound for 4 s and were asked to rate its intensity on a 100‐point visual scale. After completing the rating, participants were asked to recall the original word they had seen by typing their response. Phase II—Participants then completed the auditory imagery questionnaire. They were instructed to imagine each sound one at a time and rate how clearly they could hear the sounds on a scale of 1 (not at all) to 5 (very clear) (see Appendix A for full instructions). Phase III—Participants were presented with all test sounds from Phase I and an equal number of distractor sounds in a random order. They were asked, “Did you hear this sound before? Y/N” (i.e., is it old or new?) and responded by mouse click. For each sound, they then rated how pleasant and familiar it was on a 7‐point visual scale. The experimenter remained in the room throughout the experiment.

We measured accuracy and response time (RT) of word recall (Phase I), average clarity of auditory imagery (range 1–5; Phase II), recognition of sounds (Phase III), and ratings of intensity (Phase I), pleasantness, and familiarity (Phase III) of the sounds.

#### 2.1.5 Data analysis

Scores on the CAIS were averaged for each participant and used as a continuous variable in the analyses. For analyses of ratings and responses for the 16 trials, linear mixed effect models (LME) in R (R Core Team, 2013), using the lme4 package (Bates, Maechler, Bolker, & Walker, 2014) were conducted with word length as a fixed effect,^3^ and participant and sound as crossed random intercepts.^4^ Using the same package and predictors, log‐linear mixed effects models with a binomial model were conducted on measures of word recall accuracy and recognition accuracy for the 16 trials. To assess statistical significance, we used likelihood ratio tests with Chi‐square, comparing models with and without the following factors of interest (see Winter, 2013): condition, imagery group, and the interaction between condition and imagery group. We then report *t*‐values for simple effects with *p*‐values estimated with Monte Carlo simulation. Details of the models tested can be found in Supplementary Materials. Model‐predicted means and confidence intervals were calculated with the R package predict.glmm. (Winter, personal communication)

### 2.2 Results

#### 2.2.1 Auditory imagery

The mean rating on the CAIS was 3.56 (*SD* = 0.56; range 2.31–4.81).

#### 2.2.2 Memory

##### 2.2.2.1 Word recall

**Figure 2:**
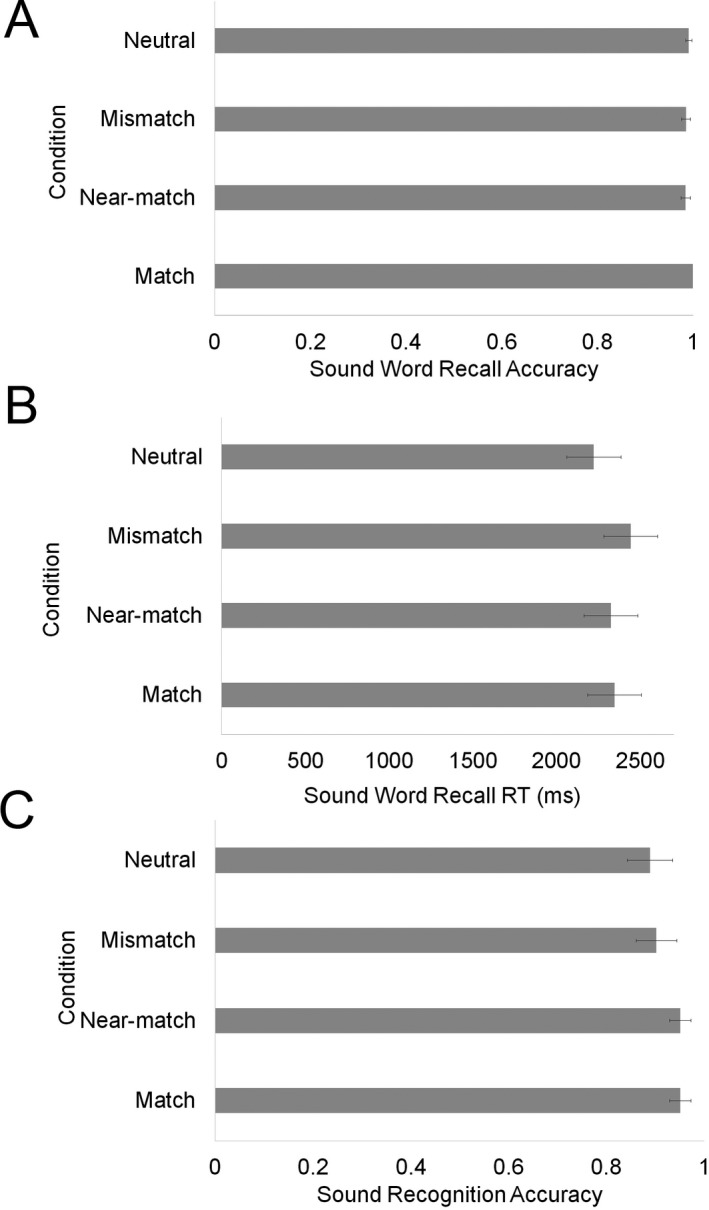
(A) Mean word recall accuracy, (B) LME predicted mean word recall response time, and (C) mean sound recognition. Error bars reflect standard error (A) and model predicted 95% confidence intervals (B and C).

Details of LME models tested for word recall accuracy can be found in Supplementary Material A1. Overall accuracy was very high (99.0%). There was no effect of condition, χ^*2*^(3) = 6.66, *p* = .08, sound imagery, χ^*2*^(1) = .70, *p* = .40, and no interaction between condition and sound imagery on accuracy, χ^*2*^(3) = 0.37, *p* = .95 (see Fig. 2A).

Word recall time was calculated as the time at which participants pressed “Enter” to submit their typed response. Word recall times outside 2.5 *SD* of a participant’s mean recall time were removed from analysis (3%). Details of LME models tested for word recall response time can be found in Supplementary Material A2. There was a significant effect of condition on word recall response time, χ^*2*^(3) = 14.46, *p* < .001.^5^ There was no effect of sound imagery, χ^*2*^(1) = .0, *p* = 1, and no interaction between condition and sound imagery on accuracy, χ^*2*^(3) = 1.20, *p* = .75. Responses in the mismatch condition were significantly longer than the neutral condition, *t* = 3.79, *p* < .001, near‐match condition, *t* = 2.09, *p* = .04, but not the match condition, *t* = 1.67, *p* = .10. The match condition was significantly different from the neutral condition, *t = *2.17, *p* = .03, but the near‐match condition was not different from the neutral condition, *t* = 1.75, *p* = .08. No other comparisons were significant. Thus, processing sounds interfered with recall of mismatch words, reflected in longer response times (see Fig. 2B^6^). Real sounds interfered with retention of mismatching words, suggesting that holding sound‐words in mind involved auditory simulation.

##### 2.2.2.2 Sound recognition

Details of the log‐linear LME models tested for sound recognition can be found in Supplementary Material A3. There was a significant effect of condition on sound recognition accuracy, χ^*2*^(3) = 14.24, *p* < .003. Recognition was significantly higher for match compared to mismatch, *z* = 3.53, *p* < .001, and neutral conditions, *z* = 4.22, *p* < .001. Similarly, near‐match was significantly higher than mismatch, *z* = 3.49, *p* < .001, and neutral, *z* = 4.09, *p* < .001. No other comparisons were significant. There was, however, no effect of sound imagery, χ^*2*^(1) = 0.23, *p* = .66. The model including the condition by imagery interaction did not converge. To summarize, words that matched or were a near‐match to the sound aided recognition memory for sounds (see Fig. 2C). This suggests mental simulation of sound at a coarse level occurred, and the overlap of activation with the real sound facilitated sound recognition.

### 2.3 Sound judgments

#### 2.3.1 Intensity ratings

**Figure 3:**
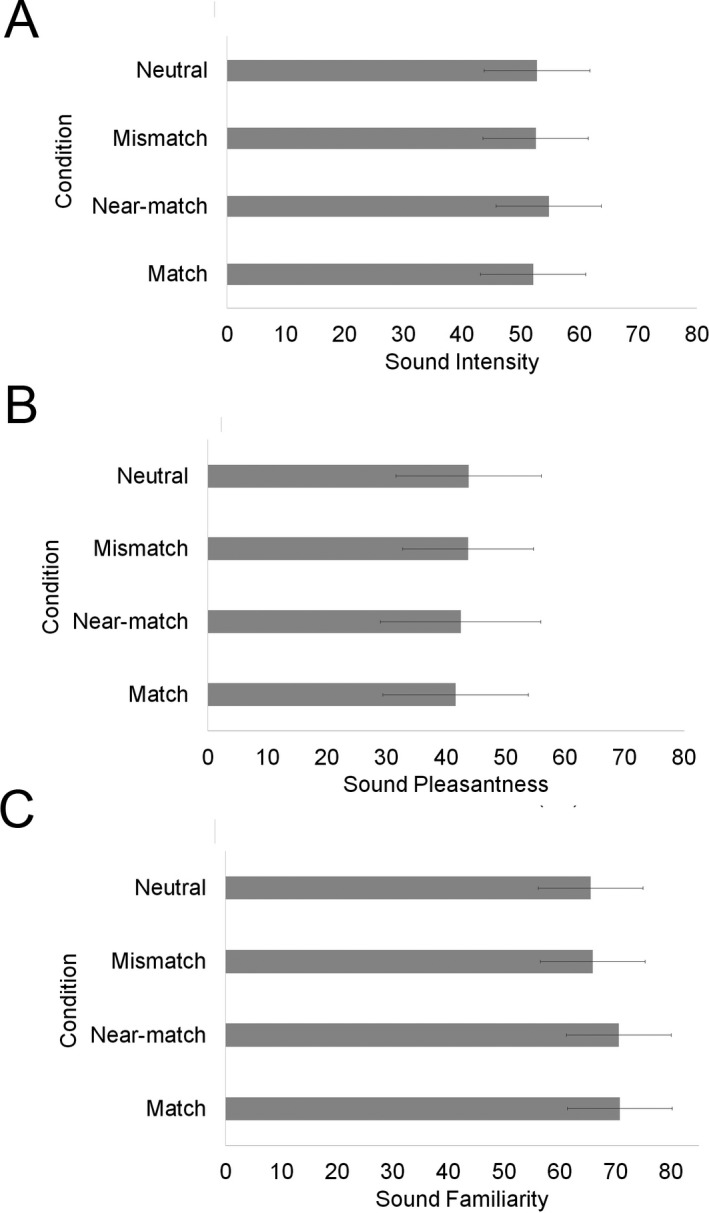
LME predicted mean (A) sound intensity rating, (B) sound pleasantness rating, and (C) sound familiarity rating. Error bars reflect 95% confidence intervals.

Details of LME models tested for sound intensity can be found in Supplementary Material A4. The model revealed no effect of condition (see Fig. 3A), χ^*2*^(3) = 2.64, *p* = .45, sound imagery score, χ^*2*^(1) = 2.55, *p* = .11, or an interaction, χ^*2*^(3) = 4.93, *p* = .18, on intensity ratings. Sound‐related words did not affect processes involved in explicit judgments of intensity.

#### 2.3.2 Pleasantness ratings

Details of LME models tested for sound pleasantness can be found in Supplementary Material A5. The model showed no effect of condition, χ^*2*^(3) = 2.80, *p* = .42 (see Fig. 3B), or sound imagery, χ^*2*^(1) = .90, *p* = .34, on pleasantness ratings, but there was a significant interaction between condition and imagery, χ^*2*^(3) = 17.14, *p* < .001. To followup the interaction, imagery was tested comparing separate models for each condition to the baseline model (including word length only). Imagery was a significant predictor of pleasantness ratings in the match condition, χ^*2*^(1) = 9.95, *p* < .001, and in the neutral condition a similar trend was observed, χ^*2*^(1) = 3.14, *p* = .08. There was no effect of imagery in the other two conditions. Higher imagery scores led to higher pleasantness ratings, but only when the sound‐word matched the sound.

#### 2.3.3 Familiarity ratings

Details of LME models tested for sound familiarity can be found in Supplementary Material A6. There was a significant effect of condition on familiarity ratings χ^*2*^(3) = 15.56, *p* = .001. Ratings were significantly higher in the match compared to mismatch, *t* = 2.75, *p* = .006, and neutral conditions, *t* = 2.97, *p* = .003. Ratings were also significantly higher in the near‐match compared to mismatch, *t* = 2.61, *p* = .009, and neutral conditions, *t* = 2.81, *p* = .005. No other comparisons between conditions were significant. Sounds were rated as more familiar with a word that matched or nearly matched the sound (see Fig. 3C), the same pattern as observed in the sound recognition task.

There was also a significant effect of imagery on familiarity ratings, χ^*2*^(1) = 14.19, *p* < .001, with higher ratings of familiarity when the imagery score was higher. But there was no interaction between imagery and condition, χ^*2*^(3) = 0.29, *p* = .96.

### 2.4 Discussion

Participants recalled words more slowly when they mismatched a heard sound. This suggests that actual sounds interfere with mental simulation of auditory information in language. Conversely, matching and near‐matching words were found to facilitate sound recognition. The two results imply, as predicted, that auditory activation from real sounds is stronger than mental simulation of auditory information, and this leads to interference of mental simulation. Mental simulation of auditory information, on the other hand, does not interfere with sound recognition but facilitates it, as shown by higher sound recognition scores for sounds paired with match and near‐match words.

There were no effects of the experimental manipulation on intensity or pleasantness ratings but there was an effect of familiarity. Sound familiarity judgments mirrored the results of sound recognition, with sounds rated as more familiar with match and near‐match words. One possible explanation for this pattern of results is that participants interpreted the familiarity question as “How familiar is this sound to you *within this experiment*?” If so, then the familiarity ratings may also be telling us about effects in memory. This is particularly likely since the familiarity rating question came after the recognition response. We also found effects involving auditory imagery. In the match condition only, higher scores on the auditory imagery scale led to higher pleasantness judgments. This effect was not predicted, but one could hypothesize that those better able to imagine sounds activated a more vivid sound representation upon reading a sound‐word. When this representation matched the real sound, this may have led to positive judgments. Yet auditory imagery ratings did not interact with condition for any other measure that did suggest mental simulation, making this explanation less likely. Higher sound imagery also predicted higher familiarity ratings. This could be expected if auditory imagery and sound recall involved similar processes; yet we did not find an effect of auditory imagery on sound recognition.

Overall, Experiment 1 verifies that the experimental paradigm is sufficient to detect effects of mental simulation. In Experiment 2, we therefore used the same paradigm for our main research goal: testing for evidence of odor simulation.

## 3 Experiment 2: Odor

### 3.1 Method

#### 3.1.1 Participants

Sixty new participants were recruited from the Radboud University Sona system (47 females, mean age = 23.98, *SD* = 6.83) and paid for their participation in shopping vouchers.

#### 3.1.2 Stimuli

The construction of stimuli paralleled Experiment 1. Sixteen target odor‐words with a near‐match, mismatch, and neutral word were used in the experiment, as described in the norming section. Sixteen odorants were used to reflect each target odor‐word, and another 16 distractor odorants were selected for the odor recognition task. Distractor odorants were chosen to reflect odor‐words chosen from the norming procedure that had not been used in the experiment. Odorants came from either real objects or essences placed in small opaque glass jars (see Appendix C for list of odorants). A thin layer of odorless white polyfiber was placed on top of the odor source so people could not see the content of the jar but could still smell it. In order to smell each odor, the lid of the jar was removed and the participant sniffed the jar.

In addition, participants also completed the Vividness of Olfactory Imagery questionnaire (VOIQ) (Gilbert, Crouch, & Kemp, 1998) between odor encoding and odor recognition. The VOIQ includes 16 items for which the participant must imagine the odor of, for example, the smell of exhaust from a passing truck, the odor of stale cigarette or cigar butts in an ashtray, and so on. As in Experiment 1, this allowed us to later examine the relationship between imagery and memory, and specifically ask whether the better ability to imagine odors might influence the retention and subsequent recognition of odors.

#### 3.1.3 Design

As before, there was one within‐participant factor with four conditions: match (odor and odor‐word match), near‐match (odor‐word for a similar odor), mismatch (different odor and odor‐word), and neutral (odor and non‐odor‐word).

#### 3.1.4 Procedure

Participants were told the experiment investigated multitasking. All individual parts of the experiment were explained to the participant before informed consent was obtained, so they were aware their memory for odors would be tested. As in Experiment 1, the experiment had three phases (see Fig. 1B).

Phase I—A trial proceeded as follows: participants were shown a word to be remembered, then were presented with an odor and asked to rate its intensity on a 100‐point visual scale. An experimenter held each open jar under the participants’ noses and instructed them to smell. After approximately 5 s the jar was removed. After rating the intensity of the odor, participants were asked to recall the original word they had seen by typing their response. The experiment continued thus until all 16 trials were completed. Phase II—Participants completed the odor imagery questionnaire (VOIQ). They were instructed to imagine each odor, one at a time, and rate how clearly they could imagine the odor on a scale of 1 (perfectly clear and as vivid as normal smell) to 5 (no image at all) (see Appendix B for full instructions). Phase III—Participants were presented with the odors from Phase I and an equal number of distractor odors in a random order, and indicated whether they had smelled the odor previously (i.e., it is “old”) or not (i.e., it is “new”) by mouse click. For each odor, after indicating whether the odor was old or new, participants also rated how pleasant and familiar it was on a 100‐point visual scale.

From this study, we were able to assess memory of both words and odors, as well as perceptual judgments of the odor stimuli. Specifically, we measured accuracy and RT of word recall (Phase I), average vividness of olfactory imagery (range 1–5; Phase II), recognition of odors (Phase III), ratings of intensity (Phase I), and pleasantness and familiarity (Phase III).

#### 3.1.5 Data analysis

Data were analyzed as in Experiment 1. Scores on the VOIQ were averaged for each participant and used as a continuous variable in the analyses.

### 3.2 Results

#### 3.2.1 Olfactory imagery

The mean rating on the VOIQ was 2.33^7^ (*SD* = 0.44; range 1.56–3.31).

#### 3.2.2 Memory

##### 3.2.2.1 Word recall

**Figure 4:**
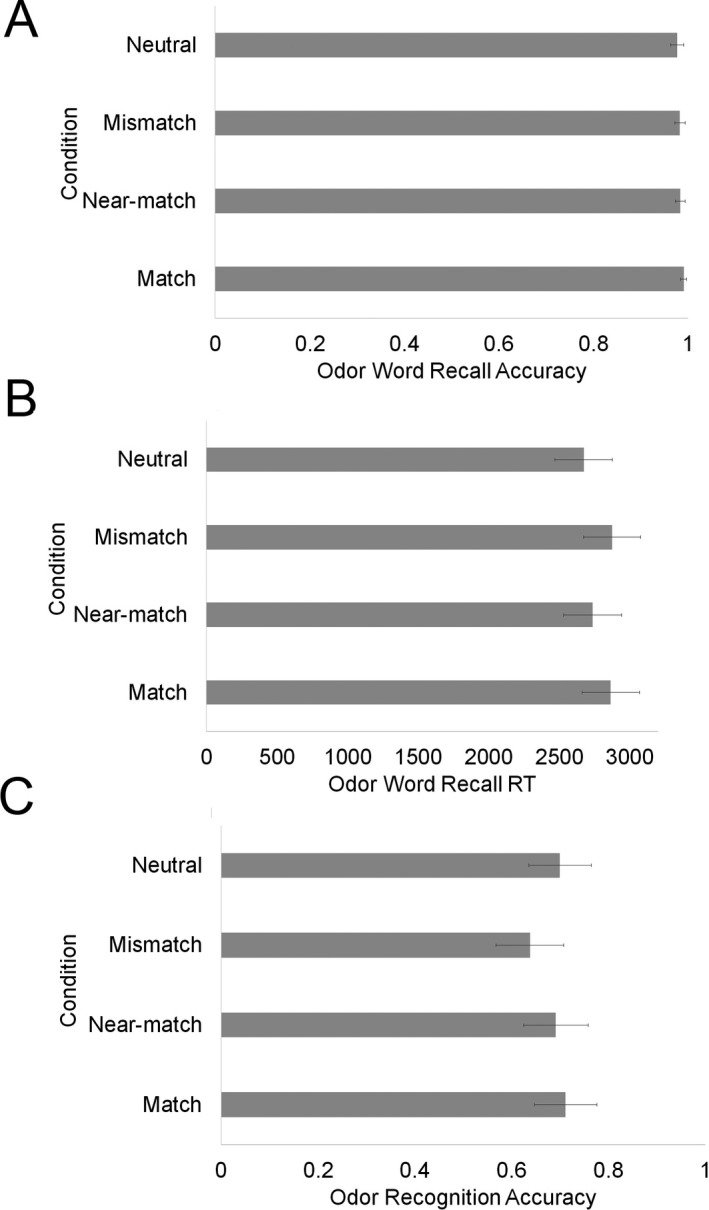
(A) LME predicted mean word recall accuracy, (B) word recall response time, and (C) odor recognition accuracy. Error bars reflect 95&percnt ; confidence intervals

Details of LME models tested for word recall accuracy can be found in Supplementary Material B1. Overall word recall accuracy was very high (97.6%) (see Fig. 4A). There was no effect of condition, χ^*2*^(3) = 2.13, *p* = .55, and no effect of imagery χ^*2*^(1) = 1.14, *p* = .29. There was, however, a significant interaction between condition and imagery, χ^*2*^(3) = 8.02, *p* = .05. The interaction was followed up by testing the effect of imagery in separate models for each condition, compared to a baseline model containing only word length.^8^ Odor imagery was a significant predictor of word recall only for words in the neutral condition, χ^*2*^(1) = 6.8, *p* = .009. Imagery did not affect recall of odor‐related words.

Again, word recall time was calculated as the time at which participants pressed “Enter” to submit their typed response. Details of LME models tested for word recall response time can be found in Supplementary Material B2. Word recall times outside 2.5 SD of a participants’ mean recall time were removed from the analysis (3%). There was no effect of condition, χ^*2*^(3) = 4.57, *p* = .21 (see Fig. 4B), no effect of imagery, χ^*2*^(3) = .40, *p* = .53, and no significant interaction between condition and odor imagery score χ^*2*^(3) = 0.61, *p* = .89.

##### 3.2.2.2 Odor recognition

Overall accuracy in the recognition test was 68%. Details of the log‐linear LME models tested for odor recognition can be found in Supplementary Material B3. There was no effect of condition on odor recognition, χ^*2*^(3) = 3.42, *p* = .33 (see Fig. 4C). The model including the interaction did not converge. Words with olfactory associations did not affect recognition of real odors, suggesting mental simulation of odor did not occur. There was, however, an effect of imagery, χ^*2*^(1) = 4.07, *p* = .04. Those better able to imagine odors had higher odor recognition scores.

### 3.3 Odor judgments

#### 3.3.1 Intensity ratings

**Figure 5:**
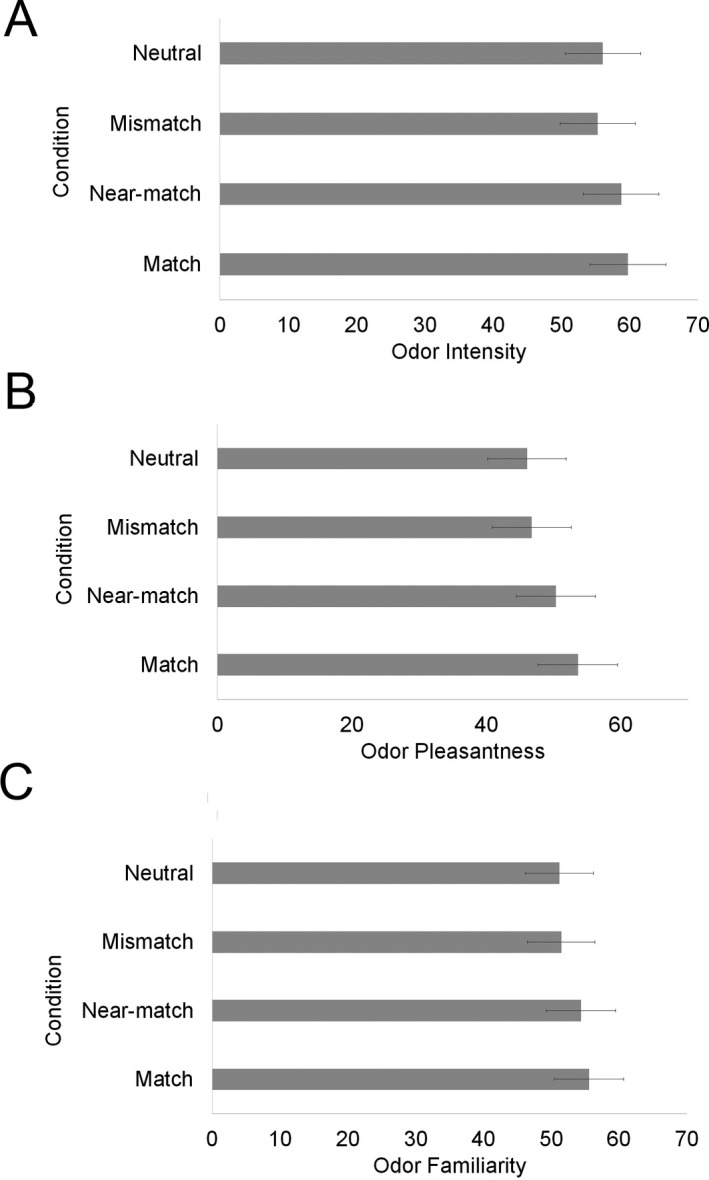
LME predicted mean (A) odor intensity ratings, (B) odor pleasantness ratings, and (C) odor familiarity ratings. Error bars denote 95&percnt ; confidence intervals.

Details of LME models tested for odor intensity can be found in Supplementary Material B4. The model revealed a main effect of condition on intensity ratings, χ^*2*^(3) = 9.68, *p* = .02, but there was no effect of odor imagery χ^*2*^(1) = 18, *p* = .67, and no interaction between condition and odor imagery χ^*2*^(3) = 3.86, *p* = .28. Participants judged odors to be more intense in the match condition than in the neutral condition, *t* = 2.14, *p* = .03, and the mismatch condition, *t* = 2.62, *p* = .009, and more intense in the near‐match condition than the mismatch condition, *t* = 2.05, *p* = .04. No other comparisons were significant (see Fig. 5A). So words that matched the odor made the odor appear more intense. Since near‐match did not differ from match but did differ from mismatch, this suggests words for similar odors also make the odor appear intense (although near‐match did not significantly differ from neutral). Information from the matching word served to enhance the perceived intensity of the odor.

#### 3.3.2 Pleasantness ratings

Details of LME models tested for odor pleasantness can be found in Supplementary Material B5. There was a main effect of condition on pleasantness ratings, χ^*2*^(3) = 17.74, *p* < .001. Odors were rated more pleasant in the match than mismatch, *t* = 3.37, *p* < .001, and neutral conditions, *t* = 3.67, *p* < .001. There was also a significant difference between near‐match and neutral, *t* = 2.14, *p = *.03, and a trend in the same direction between near‐match and mismatch, *t* = 1.76, *p = *.08. No other comparisons were significant. So match and near‐match words led to odors being perceived as more pleasant (see Fig. 5B). This suggests information related to odor‐associated words is represented at a coarse level. There was also a significant effect of imagery, χ^*2*^(1) = 5.47, *p* = .02. Those who were better at odor imagery rated the odors as less pleasant. There was no interaction between imagery and condition χ^*2*^(3) = 268, *p* = .44.

#### 3.3.3 Familiarity ratings

Details of LME models tested for odor familiarity can be found in Supplementary Material B6. There was no effect of condition on familiarity, χ^*2*^(3) = 4.21, *p* = .24 (see Fig. 5C), no effect of odor imagery, χ^*2*^(1) = 0.87, *p* = .35, and no interaction between condition and odor imagery, χ^*2*^(3) = 4.53, *p* = .21. Thus, information related to odor‐associated words does not affect the perceived familiarity of an odor, contrary to predictions.

### 3.4 Discussion

If odor‐words activate olfactory systems, then pairing odor‐words with real odors should affect memory for the words or odors. Contrary to this prediction, word‐odor pairings had no influence on memory of odors, unlike that observed for sounds and sound‐related words in Experiment 1. Perceptual judgments of intensity and pleasantness (but not familiarity) were affected. Odors were rated as more intense when paired with a matching word, and more pleasant when paired with a match or near‐match word. In terms of odor imagery, we found that participants who scored higher on the imagery scale were more accurate in recalling neutral words (unrelated to odor). It is possible that, in addition to odor imagery, the VOIQ is also a good gauge of mental imagery more generally, therefore able to predict performance for other word types. Other sensory imagery scales, such as the Plymouth Sensory Imagery Questionnaire (Andrade, May, Deeprose, Baugh, & Ganis, 2014), find strong correlations between all sensory scales. Since odor imagery did not affect recall of odor‐related words, this suggests there was difficulty activating olfactory information when reading the words. We did, however, find that odor imagery predicted odor recognition, supporting the idea that odor imagery and odor memory rely on similar systems (Lyman & McDaniel, 1990; Rinck, Rouby, & Bensafi, 2008). We also found that higher odor imagery scores led to lower pleasantness ratings. This finding was not expected and is difficult to explain. However, it suggests there may be fundamental differences in how odors are perceived depending on imagery ability.

People are sensitive to the congruency between odor and word, as witnessed by shifts in perceived intensity and pleasantness judgments. But any effects of odor‐words that do occur do not involve processes used in odor memory. We therefore did not find evidence of mental simulation of odor, but possibly an effect at a lexical‐semantic level instead.

## 4 General discussion

Our results provide evidence that sounds are mentally simulated when reading sound‐words, but they suggest that odor is not mentally simulated when reading odor‐words. Smelling odors while remembering odor‐words did not affect memory for odors. Similarly, odors did not interfere with recall of odor‐words. In the same paradigm, however, memory effects were observed when combining sound‐words with actual sounds. Sound‐words were recalled more slowly (reflecting difficulty in retrieval) when they mismatched the sound participants heard. At the same time, sounds paired with a match or near‐match word were recognized more accurately than sounds paired with mismatch or neutral words. Taken together, these results illustrate a fundamental difference in how odor versus sound‐related words are processed.

Further evidence of differential processing of odor‐ and sound‐words comes from the intensity, pleasantness, and familiarity ratings. When actual odors were paired with match and near‐match words, they were judged to be more intense and pleasant. No such effects were found for sounds. Sound‐words did not affect judgments of sound intensity or pleasantness, although (unlike odors) sound‐words did affect judgments of familiarity.

The fact that odor‐words affected judgments of odor pleasantness and intensity confirms findings from previous studies in which ratings of intensity and pleasantness of odors increased when an odor name was given (e.g., Distel & Hudson, 2001; Ferdenzi et al., 2016) or when participants were able to accurately name the odor themselves (e.g., Distel & Hudson, 2001). The present results go beyond the previous research in two ways. First, the words presented to participants were not presented as labels for the odors (i.e., participants were not told that the words and odors were related). Second, and more interestingly, intensity and pleasantness judgments are affected not only when words veridically label an odor, but also when words are merely similar to the odor. This parallels errors observed in odor naming, where people cannot give the correct name and instead produce a near‐match (Cain, de Wijk, Lulejian, Schiet, & See, 1998; Huisman & Majid, in press).

### 4.1 Localizing the effect of odor‐ and sound‐words

Experiments 1 and 2 show that sound‐ and odor‐words affect processing of sounds and odors in different ways. We had predicted that if sound/odor‐related words activated auditory/olfactory systems at the same time a real sound/odor was perceived, then this overlap in activation would affect memory for sounds/odors. We found higher recognition accuracy for sounds in the match and near‐match condition, and therefore conclude that auditory simulation occurred at a perceptual level, thereby enhancing the memory trace for the sound. We did not, however, observe similar effects in odor memory, suggesting odor‐words did not activate the olfactory system sufficiently to interfere with odor memory. Taken together, these results suggest that odor‐words are not mentally simulated in the olfactory system. However, before accepting this conclusion, we must consider the evidence in more detail.

Our results showed that odor‐words influenced ratings of intensity and pleasantness. These results could perhaps be interpreted as the result of simulation. It has been argued that odors are primarily perceived in terms of pleasantness (e.g., Yeshurun & Sobel, 2010), and odor (and taste) words have been found to be more emotionally valenced than words associated with the other perceptual modalities (Winter, 2016), so it follows pleasantness would be an important feature of mental simulation for this perceptual modality. In line with this, Bensafi, Sobel, and Kahn (2007b) found hedonic‐specific activity in piriform cortex during odor imagery. But if such simulation is occurring, it is hard to explain why odor‐words did not affect memory in Experiment 2.

Olfactory researchers consider ratings of intensity, pleasantness, and familiarity to be perceptual measures, but, of course, these ratings reflect perceptual *judgments* and so do not necessarily reflect activity in modality‐specific regions of the brain (i.e., olfactory cortex). In line with this, odor pleasantness and intensity judgments have been found to activate the orbitofrontal cortex, but not the piriform cortex (primary olfactory cortex) (Royet, Plailly, Delon‐Martin, Kareken, & Segebarth, 2003; Zatorre, Jones‐Gotman, & Rouby, 2000). It has been suggested that olfactory cognition depends on semantic, rather than perceptual, representations (e.g., Stevenson & Case, 2004), and many findings support this. For example, odor intensity and pleasantness judgments are affected by semantic information (e.g., labels) (Bensafi, Rinck, Schaal, & Rouby, 2007a; Distel & Hudson, 2001; Ferdenzi et al., 2013). Furthermore, Seubert, Freiherr, Frasnelli, Hummel, and Lundström (2013) concluded that odor identification involves retrieval of a verbal label rather than odor representations—again suggesting a reliance on semantic rather than perceptual representations.

Given this, the effects of olfactory words on judgments of intensity and pleasantness could occur at a lexico‐semantic level, such as that proposed in Vigliocco, Vinson, Lewis, and Garrett's (2004) Featural and Unitary Semantic Space Hypothesis (FUSS). In this account, a semantic level separate from modality‐specific information is postulated, and this is where featural information is bound together (Vigliocco et al., 2004; see also Plaut, 2002, for a graded account of modality‐specificity in semantics). If comprehension of odor‐words does not involve odor simulation, other methods may be used to ground meaning when describing odors, such as gesture or metaphorical language (Majid, 2013). That is, if a comprehender has difficulty conjuring the relevant odor information from odor‐words, the speaker could use gesture to highlight some quality of the odor (e.g., facial expression for negative odors) or describe the odor with a metaphor (e.g., *sweet smell*), to aid comprehension.

If the present odor results do indicate a lexical‐semantic effect and not mental simulation of odor, what can explain the González et al. (2006) finding that odor‐related words activated the olfactory cortex? We see three possibilities concerning the differences between the two studies: the linguistic stimuli, the comprehension processes involved, and sensitivity to detect simulation.

First, this study used nouns, whereas González et al. (2006) used a combination of nouns and adjectives. Adjectives that specifically describe olfactory properties (e.g., *fetid*) may be more likely to involve low‐level olfactory simulation than nouns referring to objects with some olfactory association (e.g., *cinnamon*). Supporting this, Goldberg, Perfetti, and Schneider (2006) found activation in medial orbitofrontal cortex (not piriform cortex) to fruit nouns (e.g., *peach*,* apricot*). The present set of words may also be more multimodal: as well as having strong olfactory associations, they can also have strong associations in other perceptual modalities. For example, the word *mango* may have strong taste and color associations in addition to odor associations, which could be more salient in mental simulation. One could consult lexical measures such as modality exclusivity norms to address such questions (Lynott & Connell, 2009, 2013; Speed & Majid, 2017; Winter, 2016). When looking at our own ratings (Speed & Majid, 2017), odor‐words used in this study were in fact more multimodal than sound‐words (*M* = 0.37 vs. *M* = 0.45; 0 being completely multimodal, 1 being completely unimodal), *t*(93) = 4.21, *p* < .001, *d* = .87. This is initial evidence that words strongly associated with odor are more multimodal than words association with sound.

Second, there may also be comprehension differences between González et al. (2006) and this study. In González et al. (2006), participants were instructed to read words, with a 3 s pause between each word. This may have led to deeper processing of word meaning, or even encouraged olfactory mental imagery (cf., Djordjevic et al., 2005). Furthermore, olfactory words were presented in blocks of 10, over 30 s, which could lead to increased activation of olfactory representations. In this study, participants were multitasking during word retention, and so each individual word had to be processed anew. It is possible then that a task that requires deeper semantic processing, such as making semantic judgments on words, could lead to activation of olfactory information (e.g., see Lebois, Wilson‐Mendenhall, & Barsalou, 2015).

It is also possible that the words used in this experiment are particularly poorly associated with odor information. When we designed our stimuli, we took into account similarity ratings of word meaning, but not ratings of how well each word matched the odor. We did this because if, as we propose, odor language is poorly linked with olfaction, then performance on such a rating task would be susceptible to the same problem. However, for particularly familiar odors that are easily named, it could be the case that odor information is more easily accessed from the corresponding word, making odor simulation possible.

Finally, it has to be considered that our measures of recognition were not sensitive enough to detect mental simulation of odor. Although difficult, a task in which reaction times are measured may be powerful enough to reveal effects of olfactory simulation. Note, however, that in the same paradigm, we do find evidence for auditory simulation, so this would suggest auditory simulation is stronger (i.e., leads to greater activation of the auditory system) than olfactory simulation. Our study thus provides novel evidence that simulation processes may be different in kind across sensory modalities. We also note that mental imagery scores were higher for sounds than for odors, suggesting that olfactory information is difficult to access even when engaged in explicit mental imagery.

### 4.2 Granularity of simulation

We manipulated the relationship between words and odors/sounds to be a match, near‐match, and mismatch or unrelated, in order to assess the granularity of simulation: how much detail of olfactory and auditory information is simulated. In both experiments, there was no difference between match and near‐match conditions. This suggests that odor‐words and sound‐words (regardless of the level of effect) activate information at a somewhat coarse level, reflecting odor/sound categories, rather than specific objects. However, odor‐ and sound‐words do not just activate a general olfactory/auditory representation, since there were differences between match and mismatch conditions. This suggests that simulated perceptual information (of sound) and information at a lexical‐semantic level (related to odor) contain similar levels of specificity. One reason for coarse representations of odors could be due to the way we talk about olfaction. In Western languages, there are no words to distinguish between the smell quality of peach and mango, for example (unlike some languages, cf. Majid & Burenhult, 2014; Wnuk & Majid, 2014). If there are no words to distinguish between such odor qualities (beyond broad categories such as *fruit*), then they would not be distinguished further at a semantic level. For sounds, although we can distinguish some auditory qualities such as pitch, timbre, tempo, and volume, it is also possible that fine‐grained distinctions between auditory objects are difficult. For example, it would be difficult to describe the difference between the growl of a large dog and the growl of a wolf. Therefore, constraints on the referential potential of auditory language may similarly lead to a coarse level of representation for mental simulation.

### 4.3 Contextual effects

In order to better understand the possible differences between olfactory and auditory simulations, it is worth considering what role context might play. Recent embodied language processing accounts have emphasized the important role of context in mental simulation (e.g., Lebois et al., 2015; Zwaan, 2014). Activation of information in specific modalities is more or less active depending on the context. For example, words with strong visual associations are responded to faster in a visual lexical decision task because visual attention is engaged, whereas words with strong auditory associations are faster in a naming task which engages auditory attention (Connell & Lynott, 2014). Similarly, action and visual properties are differentially activated according to task context. Van Dam, van Dijk, Bekkering, and Rueschemeyer (2012) gave participants words with strong action and color associations (e.g., *tennis ball*,* boxing glove*) and asked them to perform a go–no‐go task responding either to words associated with the color green or to words associated with foot actions. Words activated motor areas of the brain; but only when the task required judging action properties, not color properties. Given this, we might expect greater effects of olfactory associations in a context in which odor is more relevant (e.g., a cooking context).

Mental simulation can also be affected by specific experiences of individuals; therefore, simulation of odor ought to be more salient if participants have specific olfactory‐related experiences, such as that of chefs, perfumers, or vinologists, or in cultures where olfaction is particularly important (see Burenhult & Majid, 2011; O'Meara & Majid, 2016). This is in line with evidence elsewhere showing experience influences comprehension: Experience playing and watching sport affects comprehension of sports language (Beilock, Lyons, Mattarella‐Micke, Nusbaum, & Small, 2008), and piano experience affects comprehension of descriptions of pitch (Wolter, Dudschig, & Kaup, 2016). Auditory simulation may in general be more salient than olfactory simulation because audition is more relevant during everyday interactions (San Roque et al., 2015). Furthermore, the way in which language is used to describe odor may be important for the mental simulation of odor. In the West, we mostly describe odors in terms of sources (e.g., *rosemary*,* sage*), which are likely to have strong associations with information in other perceptual modalities (i.e., vision, taste). However, speakers of some languages have a dedicated olfactory lexicon, with specific terms that uniquely refer to olfactory information (e.g., Majid & Burenhult, 2014; Wnuk & Majid, 2014). It is possible that such words can more easily link to olfactory information.

## 5 Conclusion

This is the first study to specifically assess the mental simulation of odor with a behavioral experiment. We found odor‐related words affect judgments of odor quality if they denote the same or a similar odor: odor semantics is thus represented coarsely. We did not, however, observe effects of odor‐words on measures of odor memory. Our results, therefore, provide no conclusive evidence for the mental simulation of odor, in contrast to the robust evidence supporting visual and auditory simulation reported elsewhere. As such, the study sheds further light on the difficulties of odor language in Western cultures and makes clear that assessing mental simulation in less dominant modalities is critical for the development and fine‐tuning of embodied theories.

## Supporting information


**Data S1.** Details of LME models tested for all analyses

## Notes

1 Note that we use the term “mismatch” to indicate a sound mismatch, and “neutral” to indicate sound‐neutral.

2 The modality ratings here belong to a larger set of modality ratings (Speed & Majid, 2017) for which data collection continued after this study was conducted.

3 Results did not change when including word frequency also, but since some models did not converge when including them, we choose to report only the models without.

4 In all analyses, except for Word Recall RT, models did not converge for random slopes.

5 We note that when including random slopes for condition by participant and item, the effect of condition was *χ* ^*2*^(3) = 5.77, *p* = .12.

6 Due to the small variability in word recall accuracy, LME predicted means and confidence intervals could not be predicted with the predict.glmm function (Winter, personal communication).

7 Note that we reversed the scale of the VOIQ to be comparable to the CAIS, that is, 1 (no image at all) to 5 (perfectly clear and as vivid as normal smell).

8 In the match condition, the model did not converge including “length” so it was removed, leaving the baseline model with only the random intercepts.

## Appendix A CAIS Instructions

### Dutch version

#### Vragenlijst Gehoor Beeldspraak

Stelt u zich de geluiden uit de onderstaande lijst één voor één voor. Subjectief gezien, hoe duidelijk hoort u de geluiden van…

(1 = Helemaal niet; 5 = Heel duidelijk):

### English version

#### Auditory Imagery Questionnaire

Imagine the sounds listed below one at the time. Subjectively, how clearly do you hear the sounds of…

(1 = not at all; 5 = very clear):

## Appendix B VOIQ Instructions

### Dutch version

#### Levendigheid van Reukbeeldspraak Vragenlijst (LvRV)

De vragenlijst bestaat uit vier onderdelen. In elk onderdeel krijgt u een beschrijving van een situatie, gevolgd door vier uitspraken die te maken hebben met de beschreven situatie.

Sluit na het lezen van iedere vraag alstublieft uw ogen om een mentaal beeld te creëren van hoe het beschreven object of de beschreven situatie zou RUIKEN. Zodra u een mentaal beeld van deze GEUR hebt gevormd, open dan uw ogen om het gevormde mentale beeld te beoordelen. U doet dit iedere keer als u wordt gevraagd een mentaal beeld te creëren op basis van GEUR.

Geef uw beoordelingen alstublieft in de daarvoor bestemde vakjes.

Beoordelingsschaal:

1. Zeer duidelijk en zo levendig als een normale geur.
2. Duidelijk en redelijk levendig.
3. Enigszins duidelijk en levendig.
4. Vaag en onduidelijk.
5. Totaal geen beeld (slechts “het besef” dat u denkt aan het object of de situatie).

### English version

#### Vividness of Olfactory Imagery Questionnaire (VOIQ)

The questionnaire contains four sections. In each section, you will be given a description of a scene followed by four statements related to the scenario given. After reading each question, please close your eyes to construct a mental image of how the described object or scene would SMELL. Once your image of this SMELL has been formed, open your eyes to rate the mental image you constructed. You will do this for each SMELL‐based mental image requested. Please place your rating in the box provided.

Rating scale:

1. Perfectly clear and as vivid as normal smell.
2. Clear and reasonably vivid.
3. Moderately clear and vivid.
4. Vague and dim.
5. No image at all (only “knowing” that you are thinking of the object).

## Appendix C Odors used in Experiment 2

### C.1

**Table:**

Odor	Product
Afwasmiddel (detergent)	AH Puur Witpoeder
Cacao (cocoa)	Blooker Cacao
Menthol (menthol)	Erica, Pure essentiële mint olie
Lijm (glue)	Kids Glue, Panduro hobby
Cider (cider)	Magners Irish Cider
Jasmijn (jasmine)	Erica, Jasmijn olie
Eucalyptus (eucalyptus)	Erica, Pure essentiële eucalyptus olie
Venkel (fennel)	Erica, Pure essentiële venkel olie
Karamel (caramel)	Dr. Oetker, Puddingpoeder voor kloppudding
Knoflook (garlic)	AH Scherpe knoflook
Patchouli (patchouli)	Erica, Pure essentiële patchouli olie
Salie (sage)	Erica, Pure essentiële salie olie
Koriander (coriander)	Verstegen, korianderzaad
Nootmuskaat (nutmeg)	AH Gemalen nootmuskaat
Stroop (syrup)	Van Gilse, De Originele Suikerstroop
Gember (ginger)	AH verse gember/ GO TAN Gember
Parmezaan (parmesan)	Zanetti, formaggi parmigiano reggiano
Perzik (peach)	Del Monte Quality, perziken in schijven op lichte siroop
Chai (chai)	Celestial Seasonings, Chai Tea
Kaneel (cinnamon)	Verstegen, gemalen kaneel
Peterselie (parsley)	Erica, Peterselie olie
Rozemarijn (rosemary)	AH Gebroken Rozemarijn
Terpentijn (turpentine)	Hema terpentijn
Bouillon (broth)	Knorr Basis bouillon blokjes
Sinaasappel (orange)	AH Verse sinaasappel
Verf (paint)	Pebeo, gouache tempera prima color
Peper (pepper)	AH Basic, Black pepper
Mosterd (mustard)	AH Basic, Mustard
Whisky (whisky)	The Famous Grouse
Zoethout/drop (liquorice)	AH Basic, Sweet soft liquorice
Dennennaalden (pine)	Erica, Pure essentiële dennennaalden olie
Mierikswortel (horseradish)	Kühne, fijngeraspte mierikswortel
