# Supplementary material for: An Exception to Mental Simulation: No Evidence for Embodied Odor Language
Source: Cogn Sci. 2018 Feb 14;42(4):1146–78. doi: 10.1111/cogs.12593 (PMC6001635; doi:10.1111/cogs.12593)
Supplement: Supplementary file 1 — Data S1. Details of LME models tested for all analyses [file COGS-42-1146-s001.docx]

**Supplementary Material A.**

**Experiment 1 Summary of mixed effects model**

**A1. Word recall accuracy**

A1a. *Model1.lmer = glmer(RecallAccuracy ~ Length + (1|Subject) + (1|Sound), Data, family=binomial)*

Random effects Fixed effects

| **Group** | **Var** | ***SD*** |
| --- | --- | --- |
| Subject | 0 | 0 |
| Sound | 0 | 0 |

| **Variable** | **Coeff. Est.** | ***SE*** | ***z*** | ***p*** |
| --- | --- | --- | --- | --- |
| Length | 0.18 | 0.16 | 1.10 | 0.27 |

A1b. *Model2.lmer = glmer(RecallAccuracy ~ condition + Length + (1|Subject) + (1|Sound), Data, family=binomial)*

Unable to estimate parameters due to low variability across condition

A1c. *Model3.lmer = glmer(RecallAccuracy ~ Imagery + Length + (1|Subject) + (1|Sound), Data, family=binomial)*

Random effects Fixed effects

| **Group** | **Var** | ***SD*** |
| --- | --- | --- |
| Subject | 0 | 0 |
| Sound | 0 | 0 |

| **Variable** | **Coeff. Est.** | ***SE*** | ***z*** | ***p*** |
| --- | --- | --- | --- | --- |
| Imagery | 0.48 | 0.58 | 0.83 | 0.41 |
| Length | 0.17 | 0.16 | 1.09 | 0.28 |

A1d. *Model4.lmer = glmer(RecallAccuracy ~ condition + Imagery + Length +(1|Subject) + (1|Sound), Data, family=binomial)*

Unable to estimate parameters due to low variability across condition

A1e. *Model5.lmer = glmer(RecallAccuracy ~ condition*Imagery + Length + (1|Subject) + (1|Sound), Data, family=binomial)*

Unable to estimate parameters – not enough variability

**A2. Word Recall Response Times**

A2a. *Model1.lmer = lmer(WordRecallRT ~ Length + (1|Subject) + (1|Sound), Data, REML=FALSE)*

Random effects

| **Group** | **Var** | ***SD*** |
| --- | --- | --- |
| Subject | 306812.5 | 553.91 |
| Sound | 523.2 | 621.89 |

| **Variable** | **Coeff. Est.** | ***SE*** | ***t*** | ***p*** |
| --- | --- | --- | --- | --- |
| Length | 205.90 | 9.18 | 22.44 | < .001 |

Fixed effects

A2b. *Model2.lmer = lmer(WordRecallRT ~ condition + Length + (1|Subject) + (1|Sound), Data, REML=FALSE)*

Random effects

| **Group** | **Var** | ***SD*** |
| --- | --- | --- |
| Subject | 306964.8 | 554.04 |
| Sound | 741.8 | 27.24 |

Fixed effects

| **Variable** | **Coeff. Est.** | ***SE*** | ***t*** | ***p*** |
| --- | --- | --- | --- | --- |
| Length | 212.02 | 9.31 | 22.78 | < .001 |
| Condition: |  |  |  |  |
| Match vs. near-match | 24.00 | 56.77 | 0.42 | 0.67 |
| Match vs. mismatch | 95.50 | 57.22 | 1.67 | 0.10 |
| Match vs. neutral | 124.04 | 57.11 | 2.17 | 0.03 |
| Near-match vs. mismatch | 119.49 | 57.12 | 2.09 | 0.04 |
| Near-match vs. neutral | 100.05 | 57.16 | 1.75 | 0.08 |
| Mismatch vs. neutral | 219.54 | 57.87 | 3.79 | < .001 |

A2c. *Model3.lmer = lmer(WordRecallRT ~ Imagery + Length + (1|Subject) + (1|Sound), Data, REML=FALSE)*

Random effects

| **Group** | **Var** | ***SD*** |
| --- | --- | --- |
| Subject | 305891.8 | 553.07 |
| Sound | 528.7 | 22.99 |

Fixed effects

| **Variable** | **Coeff. Est.** | ***SE*** | ***t*** | ***p*** |
| --- | --- | --- | --- | --- |
| Imagery | 54.26 | 132.00 | 0.41 | 0.68 |
| Length | 205.88 | 9.18 | 22.43 | < .001 |

A2d. *Model4.lmer = lmer(WordRecallRT~ condition + Imagery + Length +(1|Subject) + (1|Sound), Data, REML=FALSE)*

Random effects

| **Group** | **Var** | ***SD*** |
| --- | --- | --- |
| Subject | 306056.6 | 553.22 |
| Sound | 746.7 | 27.33 |

Fixed effects

| **Variable** | **Coeff. Est.** | ***SE*** | ***t*** | ***p*** |
| --- | --- | --- | --- | --- |
| Length | 211.99 | 9.31 | 22.78 | < .001 |
| Imagery | 53.89 | 131.95 | 0.41 | 0.68 |
| Condition: |  |  |  |  |
| Match vs. near-match | 24.0 | 56.77 | 0.42 | 0.67 |
| Match vs. mismatch | 95.47 | 57.23 | 1.67 | 0.10 |
| Match vs. neutral | 124.05 | 57.11 | 2.17 | 0.03 |
| Near-match vs. mismatch | 119.47 | 57.12 | 2.09 | .04 |
| Near-match vs. neutral | 100.05 | 57.16 | 1.75 | 0.08 |
| Mismatch vs. neutral | 215.52 | 57.87 | 3.79 | < .001 |

A2e. *Model5.lmer = lmer(WordRecallRT ~ condition*Imagery + Length + (1|Subject) + (1|Sound), Data, REML=FALSE)*

Random effects

| **Group** | **Var** | ***SD*** |
| --- | --- | --- |
| Subject | 306194.7 | 553.30 |
| Sound | 829.2 | 28.00 |

Fixed effects

| **Variable** | **Coeff. Est.** | ***SE*** | ***t*** | ***p*** |
| --- | --- | --- | --- | --- |
| Length | 212.03 | 9.32 | 22.75 | < .001 |
| Imagery | 93.55 | 146.36 | 0.64 | 0.52 |
| Condition: |  |  |  |  |
| Match v. near-match | 27.84 | 366.91 | 0.08 | 0.94 |
| Match vs. mismatch | 344.76 | 370.88 | 0.93 | 0.35 |
| Match vs. neutral | 179.68 | 362.09 | 0.50 | 0.62 |
| Near-match vs. mismatch | 372.60 | 374.61 | 1.00 | 0.32 |
| Near-match vs. neutral | 207.52 | 366.12 | 0.57 | 0.57 |
| Mismatch vs. neutral | 165.09 | 370.08 | 0.45 | 0.66 |
| Condition*Imagery: |  |  |  |  |
| Imagery*Match vs. near-match | 1.07 | 101.98 | 0.01 | 0.99 |
| Imagery*Match vs. mismatch | 70.09 | 103.01 | 0.68 | 0.50 |
| Imagery*Match vs. neutral | 85.38 | 100.5 | 0.85 | 0.40 |
| Imagery*Near-match vs. mismatch | 71.16 | 104.07 | 0.68 | 0.49 |
| Imagery*Near-match vs. neutral | 86.45 | 101.63 | 0.85 | 0.40 |
| Imagery*Mismatch vs. neutral | 15.29 | 102.62 | 0.15 | 0.88 |

**A3. Sound recognition**

A3a. *Model1.lmer = glmer(Accuracy ~ Length + (1|Subject) + (1|Sound), Data, family=binomial)*

Random effects Fixed effects

| **Group** | **Var** | ***SD*** |
| --- | --- | --- |
| Subject | 0.72 | 0.85 |
| Sound | 0.41 | 0.64 |

| **Variable** | **Coeff. Est.** | ***SE*** | ***z*** | ***p*** |
| --- | --- | --- | --- | --- |
| Length | 0.07 | 0.05 | 1.4 | 0.16 |

A3b. *Model2.lmer = glmer(Accuracy ~ condition + Length + (1|Subject) + (1|Sound), Data, family=binomial)*

Random effects

| **Group** | **Var** | ***SD*** |
| --- | --- | --- |
| Subject | 0.76 | 0.87 |
| Sound | 0.41 | 0.64 |

Fixed effects

| **Variable** | **Coeff. Est.** | ***SE*** | ***z*** | ***p*** |
| --- | --- | --- | --- | --- |
| Length | 0.04 | 0.05 | 0.86 | 0.39 |
| Condition: |  |  |  |  |
| Match vs. near-match | 0.02 | 0.32 | 0.05 | 0.96 |
| Match vs. mismatch | 0.70 | 0.29 | 2.43 | 0.02 |
| Match vs. neutral | 0.83 | 0.29 | 2.91 | 0.003 |
| Near-match vs. mismatch | 0.72 | 0.29 | 2.47 | 0.02 |
| Near-match vs. neutral | 0.85 | 0.29 | 2.90 | 0.003 |
| Mismatch vs. neutral | 0.13 | 0.26 | 0.51 | 0.61 |

A3c. *Model3.lmer = glmer(Accuracy ~ Imagery + Length + (1|Subject) + (1|Sound), Data, family=binomial)*

Random effects

| **Group** | **Var** | ***SD*** |
| --- | --- | --- |
| Subject | 0.71 | 0.84 |
| Sound | 0.41 | 0.64 |

Fixed effects

| **Variable** | **Coeff. Est.** | ***SE*** | ***z*** | ***p*** |
| --- | --- | --- | --- | --- |
| Imagery | 0.13 | 0.27 | 0.49 | 0.62 |
| Length | 0.07 | 0.05 | 1.39 | 0.16 |

A3d. *Model4.lmer = glmer(Accuracy ~ condition + Imagery + Length +(1|Subject) + (1|Sound), Data, family=binomial)*

Random effects

| **Group** | **Var** | ***SD*** |
| --- | --- | --- |
| Subject | 0.75 | 0.87 |
| Sound | 0.41 | 0.64 |

Fixed effects

| **Variable** | **Coeff. Est.** | ***SE*** | ***z*** | ***p*** |
| --- | --- | --- | --- | --- |
| Length | 0.04 | 0.05 | 0.85 | 0.40 |
| Imagery | 0.14 | 0.27 | 0.50 | 0.61 |
| Condition: |  |  |  |  |
| Match vs. near-match^[[1]](#footnote-1)^ | - | - | - | - |
| Match vs. mismatch | 0.70 | 0.29 | 2.43 | 0.02 |
| Match vs. neutral | 0.83 | 0.29 | 2.91 | 0.003 |
| Near-match vs. mismatch | 0.72 | 0.29 | 2.47 | 0.01 |
| Near-match vs. neutral | 0.85 | 0.29 | 2.90 | 0.003 |
| Mismatch vs. neutral | 0.13 | 0.26 | 0.51 | 0.61 |

A3e. *Model5.lmer = glmer(Accuracy ~ condition*Imagery + (1|Subject) + (1|Sound), Data, family=binomial)*

Model did not converge

**A4. Intensity**

A4a. *Model1.lmer = lmer(VasScore ~ Length + (1|Subject) + (1|Sound), Data, REML=FALSE)*

Random effects Fixed effects

| **Group** | **Var** | ***SD*** |
| --- | --- | --- |
| Subject | 73.51 | 8.57 |
| Sound | 291.73 | 17.08 |

| **Variable** | **Coeff. Est.** | ***SE*** | ***t*** | ***p*** |
| --- | --- | --- | --- | --- |
| Length | 0.02 | 0.33 | 0.07 | 0.94 |

A4b. *Model2.lmer = lmer(VasScore ~ condition + Length + (1|Subject) + (1|Sound), Data, REML=FALSE)*

Random effects

| **Group** | **Var** | ***SD*** |
| --- | --- | --- |
| Subject | 73.57 | 8.58 |
| Sound | 291.11 | 17.06 |

| **Variable** | **Coeff. Est.** | ***SE*** | ***t*** | ***p*** |
| --- | --- | --- | --- | --- |
| Length | 0.05 | 0.33 | 0.14 | 0.89 |
| Condition: |  |  |  |  |
| Match vs. near-match | 2.64 | 1.77 | 1.49 | 0.14 |
| Match vs. mismatch | 0.42 | 1.77 | 0.24 | 0.81 |
| Match vs. neutral | 0.63 | 1.78 | 0.35 | 0.72 |
| Near-match vs. mismatch | 2.22 | 1.77 | 1.25 | 0.21 |
| Near-match vs. neutral | 2.01 | 1.79 | 1.12 | 0.26 |
| Mismatch vs. neutral | 0.21 | 1.80 | 0.11 | 0.91 |

Fixed effects

A4c. *Model3.lmer = lmer(VasScore ~ Imagery + Length + (1|Subject) + (1|Sound), Data, REML=FALSE)*

Random effects

| **Group** | **Var** | ***SD*** |
| --- | --- | --- |
| Subject | 69.47 | 8.34 |
| Sound | 291.81 | 17.08 |

Fixed effects

| **Variable** | **Coeff. Est.** | ***SE*** | ***t*** | ***p*** |
| --- | --- | --- | --- | --- |
| Imagery | 3.57 | 2.22 | 1.61 | 0.68 |
| Length | 0.02 | 0.33 | 0.05 | 0.96 |

A4d. *Model4.lmer = lmer(VasScore ~ condition + Imagery + Length + (1|Subject) + (1|Sound), Data, REML=FALSE)*

Random effects

| **Group** | **Var** | ***SD*** |
| --- | --- | --- |
| Subject | 69.54 | 8.34 |
| Sound | 291.18 | 17.06 |

Fixed effects

| **Variable** | **Coeff. Est.** | ***SE*** | ***t*** | ***p*** |
| --- | --- | --- | --- | --- |
| Length | 0.04 | 0.33 | 0.12 | 0.90 |
| Imagery | 3.57 | 2.22 | 1.61 | 0.11 |
| Condition: |  |  |  |  |
| Match vs. near-match | 2.64 | 1.77 | 1.49 | 0.14 |
| Match vs. mismatch | 0.42 | 1.77 | 0.24 | 0.81 |
| Match vs. neutral | 0.63 | 1.78 | 0.36 | 0.72 |
| Near-match vs. mismatch | 2.22 | 1.77 | 1.25 | 0.21 |
| Near-match vs. neutral | 2.01 | 1.79 | 1.12 | 0.26 |
| Mismatch vs. neutral | 0.21 | 1.80 | 0.12 | 0.91 |

A4e. *Model5.lmer = lmer(VasScore ~ condition*Imagery + Length + (1|Subject) + (1|Sound), Data, REML=FALSE)*

Random effects

| **Group** | **Var** | ***SD*** |
| --- | --- | --- |
| Subject | 69.57 | 8.35 |
| Sound | 290.91 | 17.06 |

Fixed effects

| **Variable** | **Coeff. Est.** | ***SE*** | ***t*** | ***p*** |
| --- | --- | --- | --- | --- |
| Length | 0.03 | 0.33 | 0.10 | 0.92 |
| Imagery | 3.56 | 2.95 | 1.21 | 0.23 |
| Condition: |  |  |  |  |
| Match v. near-match | 10.73 | 11.40 | 0.94 | 0.35 |
| Match vs. mismatch | 24.43 | 11.42 | 2.14 | 0.03 |
| Match vs. neutral | 14.85 | 11.40 | 1.30 | 0.19 |
| Near-match vs. mismatch | 13.70 | 11.40 | 1.20 | 0.23 |
| Near-match vs. neutral | 4.122 | 11.42 | 0.36 | 0.72 |
| Mismatch vs. neutral | 9.57 | 11.41 | 0.84 | 0.40 |
| Condition*Imagery: |  |  |  |  |
| Imagery*Match vs. near-match | 3.76 | 3.17 | 1.19 | 0.24 |
| Imagery*Match vs. mismatch | 6.99 | 3.17 | 2.20 | 0.03 |
| Imagery*Match vs. neutral | 4.36 | 3.17 | 1.38 | 0.17 |
| Imagery*Near-match vs. mismatch | 3.23 | 3.17 | 1.02 | 0.31 |
| Imagery*Near-match vs. neutral | 0.60 | 3.17 | 0.19 | 0.85 |
| Imagery*Mismatch vs. neutral | 2.63 | 3.17 | 0.83 | 0.41 |

**A5. Pleasantness**

A5a. *Model1.lmer = lmer(VasScore ~ Length + (1|Subject) + (1|Sound), Data, REML=FALSE)*

Random effects Fixed effects

| **Group** | **Var** | ***SD*** |
| --- | --- | --- |
| Subject | 24.84 | 4.94 |
| Sound | 599.58 | 24.49 |

| **Variable** | **Coeff. Est.** | ***SE*** | ***t*** | ***p*** |
| --- | --- | --- | --- | --- |
| Length | 0 | 0 | 0.03 | 0.98 |

A5b. *Model2.lmer = lmer(VasScore ~ condition + Length + (1|Subject) + (1|Sound), Data, REML=FALSE)*

Random effects

| **Group** | **Var** | ***SD*** |
| --- | --- | --- |
| Subject | 24.88 | 4.99 |
| Sound | 598.67 | 24.47 |

| **Variable** | **Coeff. Est.** | ***SE*** | ***t*** | ***p*** |
| --- | --- | --- | --- | --- |
| Length | 0.02 | 0.29 | .07 | 0.95 |
| Condition: |  |  |  |  |
| Match vs. near-match | 0.82 | 1.53 | 0.54 | 0.59 |
| Match vs. mismatch | 2.09 | 1.53 | 1.36 | 0.17 |
| Match vs. neutral | 2.17 | 1.54 | 1.41 | 0.16 |
| Near-match vs. mismatch | 1.27 | 1.53 | 0.83 | 0.41 |
| Near-match vs. neutral | 1.36 | 1.55 | 0.87 | 0.38 |
| Mismatch vs. neutral | 0.09 | 1.56 | 0.06 | 0.95 |

Fixed effects

A5c. *Model3.lmer = lmer(VasScore ~ Imagery + Length + (1|Subject) + (1|Sound), Data, REML=FALSE)*

Random effects

| **Group** | **Var** | ***SD*** |
| --- | --- | --- |
| Subject | 24.2 | 4.92 |
| Sound | 599.7 | 24.49 |

Fixed effects

| **Variable** | **Coeff. Est.** | ***SE*** | ***t*** | ***p*** |
| --- | --- | --- | --- | --- |
| Imagery | 1.42 | 1.49 | 0.95 | 0.35 |
| Length | 0.01 | 0.28 | 0.04 | 0.97 |

A5d. *Model4.lmer = lmer(VasScore ~ condition + Imagery + Length + (1|Subject) + (1|Sound), Data, REML=FALSE)*

Random effects

| **Group** | **Var** | ***SD*** |
| --- | --- | --- |
| Subject | 24.25 | 4.92 |
| Sound | 598.79 | 24.47 |

Fixed effects

| **Variable** | **Coeff. Est.** | ***SE*** | ***t*** | ***p*** |
| --- | --- | --- | --- | --- |
| Length | 0.02 | 0.29 | 0.05 | 0.96 |
| Imagery | 1.42 | 1.49 | 0.95 | 0.35 |
| Condition: |  |  |  |  |
| Match vs. near-match | 0.82 | 1.53 | 0.54 | 0.59 |
| Match vs. mismatch | 2.09 | 1.54 | 1.36 | 0.17 |
| Match vs. neutral | 2.17 | 1.54 | 1.41 | 0.16 |
| Near-match vs. mismatch | 0.09 | 1.546 | 0.06 | 0.96 |
| Near-match vs. neutral | 1.35 | 1.55 | 0.87 | 0.38 |
| Mismatch vs. neutral | 0.09 | 1.56 | 0.06 | 0.96 |

A5e. *Model5.lmer = lmer(VasScore ~ condition*Imagery + Length + (1|Subject) + (1|Sound), Data, REML=FALSE)*

Random effects

| **Group** | **Var** | ***SD*** |
| --- | --- | --- |
| Subject | 24.59 | 4.96 |
| Sound | 596.30 | 24.42 |

Fixed effects

| **Variable** | **Coeff. Est.** | ***SE*** | ***t*** | ***p*** |
| --- | --- | --- | --- | --- |
| Length | 0 | 0.29 | 0.01 | 0.99 |
| Imagery | 0.41 | 2.24 | 0.18 | 0.85 |
| Condition: |  |  |  |  |
| Match v. near-match | 32.36 | 9.81 | 3.30 | 0.001 |
| Match vs. mismatch | 37.67 | 9.83 | 3.83 | < .001 |
| Match vs. neutral | 15.87 | 9.81 | 1.62 | 0.11 |
| Near-match vs. mismatch | 5.32 | 9.81 | 0.54 | 0.59 |
| Near-match vs. neutral | 16.49 | 9.83 | 1.68 | 0.09 |
| Mismatch vs. neutral | 21.81 | 9.82 | 2.22 | 0.03 |
| Condition*Imagery: |  |  |  |  |
| Imagery*Match vs. near-match | 8.87 | 2.72 | 3.26 | 0.001 |
| Imagery*Match vs. mismatch | 10.01 | 2.73 | 3.67 | < .001 |
| Imagery*Match vs. neutral | 3.85 | 2.72 | 1.41 | 0.16 |
| Imagery*Near-match vs. mismatch | 1.14 | 2.72 | 0.42 | 0.68 |
| Imagery*Near-match vs. neutral | 5.01 | 2.73 | 1.84 | 0.07 |
| Imagery*Mismatch vs. neutral | 6.15 | 2.72 | 2.26 | 0.02 |

**A6. Familiarity**

A6a. *Model1.lmer = lmer(VasScore ~ Length + (1|Subject) + (1|Sound), Data, REML=FALSE)*

Random effects

| **Group** | **Var** | ***SD*** |
| --- | --- | --- |
| Subject | 100.40 | 10.02 |
| Sound | 315.5 | 17.76 |

Fixed effects

| **Variable** | **Coeff. Est.** | ***SE*** | ***t*** | ***p*** |
| --- | --- | --- | --- | --- |
| Length | 0.31 | 0.33 | 0.93 | 0.36 |

A6b. *Model2.lmer = lmer(VasScore ~ condition + Length + (1|Subject) + (1|Sound), Data, REML=FALSE)*

Random effects

| **Group** | **Var** | ***SD*** |
| --- | --- | --- |
| Subject | 100.8 | 10.04 |
| Sound | 316.8 | 17.79 |

| **Variable** | **Coeff. Est.** | ***SE*** | ***t*** | ***p*** |
| --- | --- | --- | --- | --- |
| Length | 0.21 | 0.34 | 0.62 | 0.54 |
| Condition: |  |  |  |  |
| Match vs. near-match | 0.26 | 1.77 | 0.14 | 0.89 |
| Match vs. mismatch | 4.88 | 1.78 | 2.75 | 0.006 |
| Match vs. neutral | 5.31 | 1.79 | 2.97 | 0.003 |
| Near-match vs. mismatch | 4.62 | 1.77 | 2.61 | 0.001 |
| Near-match vs. neutral | 5.05 | 1.80 | 2.81 | 0.005 |
| Mismatch vs. neutral | 0.43 | 1.81 | 0.24 | 0.82 |

Fixed effects

A6c. *Model3.lmer = lmer(VasScore ~ Imagery + Length + (1|Subject) + (1|Sound), Data, REML=FALSE)*

Random effects

| **Group** | **Var** | ***SD*** |
| --- | --- | --- |
| Subject | 74.22 | 8.62 |
| Sound | 315.16 | 17.75 |

Fixed effects

| **Variable** | **Coeff. Est.** | ***SE*** | ***t*** | ***p*** |
| --- | --- | --- | --- | --- |
| Imagery | 9.11 | 2.28 | 4 | < .001 |
| Length | 0.32 | 0.33 | 0.96 | 0.33 |

A6d. *Model4.lmer = lmer(VasScore ~ condition + Imagery + Length + (1|Subject) + (1|Sound), Data, REML=FALSE)*

Random effects

| **Group** | **Var** | ***SD*** |
| --- | --- | --- |
| Subject | 74.61 | 8.64 |
| Sound | 316.16 | 17.78 |

| **Variable** | **Coeff. Est.** | ***SE*** | ***t*** | ***p*** |
| --- | --- | --- | --- | --- |
| Length | 0.22 | 0.33 | 0.65 | 0.52 |
| Imagery | 9.10 | 2.28 | 4.00 | < .001 |
| Condition: |  |  |  |  |
| Match vs. near-match | 0.26 | 1.77 | 0.15 | 0.55 |
| Match vs. mismatch | 4.88 | 1.78 | 2.75 | 0.006 |
| Match vs. neutral | 5.30 | 1.79 | 2.97 | <.001 |
| Near-match vs. mismatch | 4.62 | 1.77 | 2.61 | 0.01 |
| Near-match vs. neutral | 5.04 | 1.80 | 2.81 | 0.01 |
| Mismatch vs. neutral | 0.42 | 1.81 | 0.23 | 0.82 |

Fixed effects

A6e. *Model5.lmer = lmer(VasScore ~ condition*Imagery + Length + (1|Subject) + (1|Sound), Data, REML=FALSE)*

Random effects

| **Group** | **Var** | ***SD*** |
| --- | --- | --- |
| Subject | 74.62 | 8.64 |
| Sound | 316.15 | 17.78 |

Fixed effects

| **Variable** | **Coeff. Est.** | ***SE*** | ***t*** | ***p*** |
| --- | --- | --- | --- | --- |
| Length | 0.22 | 0.34 | 0.66 | 0.51 |
| Imagery | 8.40 | 3.00 | 2.80 | .01 |
| Condition: |  |  |  |  |
| Match v. near-match | 3.17 | 11.45 | 0.28 | 0.78 |
| Match vs. mismatch | 5.67 | 11.47 | 0.49 | 0.62 |
| Match vs. neutral | 1.08 | 11.45 | 0.09 | 0.93 |
| Near-match vs. mismatch | 8.83 | 11.45 | 0.77 | 0.44 |
| Near-match vs. neutral | 4.24 | 11.48 | 0.37 | 0.71 |
| Mismatch vs. neutral | 4.59 | 11.46 | 0.40 | 0.69 |
| Condition*Imagery: |  |  |  |  |
| Imagery*Match vs. near-match | 0.96 | 3.18 | 0.30 | 0.76 |
| Imagery*Match vs. mismatch | 0.22 | 3.18 | 0.07 | 0.94 |
| Imagery*Match vs. neutral | 1.19 | 3.18 |  | 0.71 |
| Imagery*Near-match vs. mismatch | 1.18 | 3.18 | 0.37 | 0.71 |
| Imagery*Near-match vs. neutral | 0.23 | 3.19 | 0.07 | 0.94 |
| Imagery*Mismatch vs. neutral | 1.41 | 3.18 | 0.44 | 0.66 |

**Supplementary Material B.**

**Experiment 1 Summary of mixed effects models**

**B1. Word recall accuracy**

B1a. *Model1.lmer = glmer(WordRecallACC ~ Length + (1|Subject) + (1|Odor), Data, family=binomial)*

Random effects Fixed effects

| **Group** | **Var** | ***SD*** |
| --- | --- | --- |
| Subject | 0.92 | 0.96 |
| Odor | 0 | 0 |

| **Variable** | **Coeff. Est.** | ***SE*** | ***z*** | ***p*** |
| --- | --- | --- | --- | --- |
| Length | 0.07 | 0.10 | 0.68 | 0.50 |

B1b.  *Model2.lmer = glmer(WordRecallACC ~ condition + Length + (1|Subject) + (1|Odor), Data, family=binomial)*

Random effects

| **Group** | **Var** | ***SD*** |
| --- | --- | --- |
| Subject | 0 | 0.97 |
| Odor | 0 | 0 |

Fixed effects

| **Variable** | **Coeff. Est.** | ***SE*** | ***z*** | ***p*** |
| --- | --- | --- | --- | --- |
| Length | 0.03 | 0.11 | 0.31 | 0.76 |
| Condition: |  |  |  |  |
| Match vs. near-match | 0.64 | 0.76 | 0.84 | 0.40 |
| Match vs. mismatch | 0.68 | 0.73 | 0.93 | 0.35 |
| Match vs. neutral | 0.97 | 0.70 | 0.38 | 0.17 |
| Near-match vs. mismatch | 0.04 | 0.60 | 0.07 | 0.95 |
| Near-match vs. neutral | 0.33 | 0.56 | 0.59 | 0.55 |
| Mismatch vs. neutral | 0.30 | 0.11 | 0.31 | 0.76 |

B1c. *Model3.lmer = glmer(WordRecallACC ~ ImageryScore + Length + (1|Subject) + (1|Odor), Data, family=binomial)*

Random effects Fixed effects

| **Group** | **Var** | ***SD*** |
| --- | --- | --- |
| Subject | 0 | 0.90 |
| Odor | 0 | 0 |

| **Variable** | **Coeff. Est.** | ***SE*** | ***z*** | ***p*** |
| --- | --- | --- | --- | --- |
| Imagery | 0.62 | 0.58 | 1.07 | 0.28 |
| Length | 0.07 | 0.10 | 0.71 | 0.48 |

B1d. *Model4.lmer = glmer(WordRecallACC ~ condition + ImageryScore + Length +(1|Subject) + (1|Odor), Data, family=binomial)*

Random effects

| **Group** | **Var** | ***SD*** |
| --- | --- | --- |
| Subject | 0 | 0.92 |
| Odor | 0 | 0 |

Fixed effects

| **Variable** | **Coeff. Est.** | ***SE*** | ***z*** | ***p*** |
| --- | --- | --- | --- | --- |
| Length | 0.04 | 0.11 | 0.34 | 0.74 |
| Imagery | 0.62 | 0.58 | 1.06 | 0.29 |
| Condition: |  |  |  |  |
| Match vs. near-match | 0.63 | 0.76 | 0.83 | 0.41 |
| Match vs. mismatch | 0.67 | 0.73 | 0.92 | 0.36 |
| Match vs. neutral | 0.97 | 0.71 | 1.37 | 0.17 |
| Near-match vs. mismatch | 0.04 | 0.60 | 0.07 | 0.94 |
| Near-match vs. neutral | 0.34 | 0.56 | 0.60 | 0.55 |
| Mismatch vs. neutral | 0.29 | 0.56 | 0.53 | 0.60 |

B1e. *Model5.lmer = glmer(WordRecallACC ~ condition*ImageryScore + Length + (1|Subject) + (1|Odor), Data, family=binomial)*

Model did not converge

**B2. Word Recall Response Times**

B2a. *Model1.lmer = lmer(RT ~ Length + (1|Subject) + (1|Odour), RTdata, REML=FALSE)*

Random effects

| **Group** | **Var** | ***SD*** |
| --- | --- | --- |
| Subject | 486384 | 697.4 |
| Odor | 19578 | 139.9 |

| **Variable** | **Coeff. Est.** | ***SE*** | ***t*** | ***p*** |
| --- | --- | --- | --- | --- |
| Length | 208.3 | 14.25 | 14.61 | < .001 |

Fixed effects

B2b.  *Model2.lmer = lmer(RT ~ condition + Length + (1|Subject) + (1|Odour), RTdata, REML=FALSE)*

Random effects

| **Group** | **Var** | ***SD*** |
| --- | --- | --- |
| Subject | 485862 | 697 |
| Odor | 19665 | 140.2 |

Fixed effects

| **Variable** | **Coeff. Est.** | ***SE*** | ***t*** | ***p*** |
| --- | --- | --- | --- | --- |
| Length | 202.78 | 166.79 | 8.957 | < .001 |
| Condition: |  |  |  |  |
| Match vs. near-match | 48.78 | 90.06 | 0.54 | 0.59 |
| Match vs. mismatch | 13.50 | 84.66 | 0.16 | 0.87 |
| Match vs. neutral | 149.86 | 86.23 | 1.74 | 0.08 |
| Near-match vs. mismatch | 62.28 | 85.15 | 0.73 | 0.47 |
| Near-match vs. neutral | 101.08 | 84.30 | 1.20 | 0.23 |
| Mismatch vs. neutral | 163.36 | 83.57 | 1.96 | 0.05 |

B2c. *Model3.lmer = lmer(RT ~ ImageryScore + Length + (1|Subject) + (1|Odor), Data, REML=FALSE)*

Random effects

| **Group** | **Var** | ***SD*** |
| --- | --- | --- |
| Subject | 482758 | 694.8 |
| Odor | 19581 | 139.9 |

Fixed effects

| **Variable** | **Coeff. Est.** | ***SE*** | ***t*** | ***p*** |
| --- | --- | --- | --- | --- |
| Imagery | 4.88 | 7.75 | 0.63 | 0.53 |
| Length | 208.34 | 14.25 | 14.62 | < .001 |

B2d. *Model4.lmer = lmer(RT~ condition + ImageryScore + Length +(1|Subject) + (1|Odor), Data, REML=FALSE)*

Random effects

| **Group** | **Var** | ***SD*** |
| --- | --- | --- |
| Subject | 482298 | 694.5 |
| Odor | 19669 | 140.2 |

Fixed effects

| **Variable** | **Coeff. Est.** | ***SE*** | ***t*** | ***p*** |
| --- | --- | --- | --- | --- |
| Length | 202.837 | 15.48 | 13.11 | < .001 |
| Imagery | 4.84 | 7.75 | 0.63 | 0.53 |
| Condition: |  |  |  |  |
| Match vs. near-match | 48.57 | 90.07 | 0.54 | 0.59 |
| Match vs. mismatch | 13.53 | 84.66 | 0.16 | 0.87 |
| Match vs. neutral | 149.73 | 86.23 | 1.74 | 0.08 |
| Near-match vs. mismatch | 62.09 | 85.16 | 0.73 | 0.47 |
| Near-match vs. neutral | 101.16 | 84.30 | 1.2 | 0.23 |
| Mismatch vs. neutral | 163.26 | 83.57 | 1.95 | 0.05 |

B2e. *Model5.lmer = lmer(RT ~ condition*ImageryScore + Length + (1|Subject) + (1|Odor), Data, REML=FALSE)*

Random effects

| **Group** | **Var** | ***SD*** |
| --- | --- | --- |
| Subject | 482177 | 694.4 |
| Odor | 19338 | 139.1 |

Fixed effects

| **Variable** | **Coeff. Est.** | ***SE*** | ***t*** | ***p*** |
| --- | --- | --- | --- | --- |
| Length | 202.352 | 15.48 | 13.07 | < .001 |
| Imagery | 4.30 | 8.80 | 0.49 | 0.63 |
| Condition: |  |  |  |  |
| Match v. near-match | 12.57 | 123.67 | 0.10 | 0.92 |
| Match vs. mismatch | 24.00 | 121.16 | 0.20 | 0.84 |
| Match vs. neutral | 117.10 | 121.49 | 0.96 | 0.34 |
| Near-match vs. mismatch | 11.43 | 121.11 | 0.09 | 0.93 |
| Near-match vs. neutral | 129.67 | 120.13 | 1.08 | 0.28 |
| Mismatch vs. neutral | 141.1 | 120.26 | 1.17 | 0.24 |
| Condition*Imagery: |  |  |  |  |
| Imagery*Match vs. near-match | 4.95 | 6.84 | 0.73 | 0.47 |
| Imagery*Match vs. mismatch | 0.87 | 6.83 | 0.13 | 0.90 |
| Imagery*Match vs. neutral | 2.64 | 6.81 | 0.39 | 0.70 |
| Imagery*Near-match vs. mismatch | 4.08 | 6.88 | 0.59 | 0.55 |
| Imagery*Near-match vs. neutral | 2.32 | 6.86 | 0.34 | 0.74 |
| Imagery*Mismatch vs. neutral | 1.77 | 6.86 | 0.26 | 0.80 |

**B3. Odor recognition**

B3a. *Model1.lmer = glmer(Accuracy ~ Length + (1|Subject) + (1|OdorName), Data, family=binomial)*

Random effects Fixed effects

| **Group** | **Var** | ***SD*** |
| --- | --- | --- |
| Subject | 0.02 | 0.13 |
| Odor | 0.12 | 0.35 |

| **Variable** | **Coeff. Est.** | ***SE*** | ***z*** | ***p*** |
| --- | --- | --- | --- | --- |
| Length | 0.02 | 0.03 | 0.62 | 0.53 |

B3b. *Model2.lmer = glmer(Accuracy ~ condition + Length + (1|Subject) + (1|OdorName), Data, family=binomial)*

Random effects

| **Group** | **Var** | ***SD*** |
| --- | --- | --- |
| Subject | 0.02 | 0.13 |
| Odor | 0.12 | 0.35 |

Fixed effects

| **Variable** | **Coeff. Est.** | ***SE*** | ***z*** | ***p*** |
| --- | --- | --- | --- | --- |
| Length | 0.03 | 0.04 | 0.70 | 0.48 |
| Condition: |  |  |  |  |
| Match vs. near-match | 0.10 | 0.22 | 0.44 | 0.66 |
| Match vs. mismatch | 0.34 | 0.20 | 1.68 | 0.10 |
| Match vs. neutral | 0.06 | 0.21 | 0.27 | 0.79 |
| Near-match vs. mismatch | 0.24 | 0.20 | 1.21 | 0.23 |
| Near-match vs. neutral | 0.04 | 0.20 | 0.19 | 0.85 |
| Mismatch vs. neutral | 0.28 | 0.04 | 0.70 | 0.48 |

B3c. *Model3.lmer = glmer(Accuracy ~ ImageryScore + Length + (1|Subject) + (1|OdorName), Data, family=binomial)*

Random effects

| **Group** | **Var** | ***SD*** |
| --- | --- | --- |
| Subject | 0 | 0 |
| Odor | 0.12 | 0.35 |

Fixed effects

| **Variable** | **Coeff. Est.** | ***SE*** | ***Z*** | ***p*** |
| --- | --- | --- | --- | --- |
| Imagery | 0.33 | .013 | 2.03 | 0.04 |
| Length | 0.02 | 0.03 | 0.68 | 0.50 |

B3d. *Model4.lmer = glmer(Accuracy ~ condition + ImageryScore + Length +(1|Subject) + (1|OdorName), Data, family=binomial)*

Random effects

| **Group** | **Var** | ***SD*** |
| --- | --- | --- |
| Subject | 0 | 0 |
| Odor | 0 | 0 |

Fixed effects

| **Variable** | **Coeff. Est.** | ***SE*** | ***z*** | ***p*** |
| --- | --- | --- | --- | --- |
| Length | 0.03 | 0.4 | 0.76 | 0.45 |
| Imagery | 0.33 | 0.16 | 2.04 | 0.04 |
| Condition: |  |  |  |  |
| Match vs. near-match | 0.10 | 0.22 | 0.47 | 0.64 |
| Match vs. mismatch | 0.34 | 0.20 | 1.69 | 0.09 |
| Match vs. neutral | 0.06 | 0.21 | 0.28 | 0.77 |
| Near-match vs. mismatch | 0.24 | 0.20 | 1.19 | 0.23 |
| Near-match vs. neutral | 0.04 | 0.20 | 0.20 | 0.84 |
| Mismatch vs. neutral | 0.28 | 0.04 | 0.70 | 0.48 |

B3e. *Model5.lmer = glmer(Accuracy ~ condition*ImageryScore + (1|Subject) + (1|OdorName), Data, family=binomial)*

Model failed to converge

**B4. Intensity**

B4a. *Model1.lmer = lmer(VasScore ~ Length + (1|Subject) + (1|Odor), Data, REML=FALSE)*

Random effects

| **Group** | **Var** | ***SD*** | |
| --- | --- | --- | --- |
| Subject | 41.45 | 6.44 |  |
| Odor | 94.58 | 9.73 | |

Fixed effects

| **Variable** | **Coeff. Est.** | ***SE*** | ***t*** | ***p*** |
| --- | --- | --- | --- | --- |
| Length | 0.17 | 0.28 | 0.60 | 0.55 |

B4b. *Model2.lmer = lmer(VasScore ~ condition + Length + (1|Subject) + (1|Odor), Data, REML=FALSE)*

Random effects

| **Group** | **Var** | ***SD*** |
| --- | --- | --- |
| Subject | 41.72 | 6.46 |
| Sound | 94.78 | 9.74 |

Fixed effects

| **Variable** | **Coeff. Est.** | ***SE*** | ***t*** | ***p*** |
| --- | --- | --- | --- | --- |
| Length | 0.08 | 0.31 | 0.26 | 0.80 |
| Condition: |  |  |  |  |
| Match vs. near-match | 0.97 | 1.78 | 0.55 | 0.59 |
| Match vs. mismatch | 4.41 | 1.68 | 2.62 | 0.01 |
| Match vs. neutral | 3.64 | 1.70 | 2.14 | 0.03 |
| Near-match vs. mismatch | 3.43 | 1.68 | 2.05 | 0.04 |
| Near-match vs. neutral | 2.67 | 1.66 | 1.61 | 0.11 |
| Mismatch vs. neutral | 0.77 | 1.65 | 0.47 | 0.64 |

B4c. *Model3.lmer = lmer(VasScore ~ ImageryScore + Length + (1|Subject) + (1|Odor), Data, REML=FALSE)*

Random effects

| **Group** | **Var** | ***SD*** |
| --- | --- | --- |
| Subject | 41.27 | 6.42 |
| Odor | 94.58 | 9.83 |

Fixed effects

| **Variable** | **Coeff. Est.** | ***SE*** | ***t*** | ***p*** |
| --- | --- | --- | --- | --- |
| Imagery | 0.97 | 2.31 | 0.42 | 0.68 |
| Length | 0.17 | 0.29 | 0.59 | 0.56 |

B4d. *Model4.lmer = lmer(VasScore ~ condition + ImageryScore + Length + (1|Subject) + (1|Odor), Data, REML=FALSE)*

Random effects

| **Group** | **Var** | ***SD*** |
| --- | --- | --- |
| Subject | 41.53 | 6.45 |
| Odor | 94.78 | 9.74 |

Fixed effects

| **Variable** | **Coeff. Est.** | ***SE*** | ***t*** | ***p*** |
| --- | --- | --- | --- | --- |
| Length | 0.08 | 0.31 | 0.25 | 0.80 |
| Imagery | 0.99 | 2.31 | 0.25 | 0.80 |
| Condition: |  |  |  |  |
| Match vs. near-match | 0.98 | 1.78 | 0.55 | 0.58 |
| Match vs. mismatch | 4.41 | 1.68 | 2.63 | 0.01 |
| Match vs. neutral | 3.64 | 1.70 | 2.14 | 0.03 |
| Near-match vs. mismatch | 3.43 | 1.68 | 2.04 | 0.04 |
| Near-match vs. neutral | 2.67 | 2.31 | 0.43 | 0.67 |
| Mismatch vs. neutral | 0.77 | 1.65 | 0.47 | 0.64 |

B4e. *Model5.lmer = lmer(VasScore ~ condition*ImageryScore + Length + (1|Subject) + (1|Odor), Data, REML=FALSE)*

Random effects

| **Group** | **Var** | ***SD*** |
| --- | --- | --- |
| Subject | 41.63 | 6.45 |
| Odor | 95.97 | 9.80 |

Fixed effects

| **Variable** | **Coeff. Est.** | ***SE*** | ***t*** | ***p*** |
| --- | --- | --- | --- | --- |
| Length | 0.07 | 0.31 | 0.23 | 0.82 |
| Imagery | 2.35 | 3.27 | 0.72 | 0.47 |
| Condition: |  |  |  |  |
| Match v. near-match | 5.36 | 10.24 | 0.52 | 0.60 |
| Match vs. mismatch | 4.97 | 10.25 | 0.49 | 0.63 |
| Match vs. neutral | 9.49 | 10.22 | 0.93 | 0.35 |
| Near-match vs. mismatch | 10.33 | 1022 | 1.01 | 0.31 |
| Near-match vs. neutral | 14.86 | 10.25 | 1045 | 0.15 |
| Mismatch vs. neutral | 4.53 | 10.20 | 0.44 | 0.66 |
| Condition*Imagery: |  |  |  |  |
| Imagery*Match vs. near-match | 1.63 | 3.77 | 0.43 | 0.66 |
| Imagery*Match vs. mismatch | 3.51 | 3.78 | 0.93 | 0.35 |
| Imagery*Match vs. neutral | 4.92 | 3.77 | 1.31 | 0.19 |
| Imagery*Near-match vs. mismatch | 5.15 | 3.77 | 1.37 | 0.17 |
| Imagery*Near-match vs. neutral | 6.56 | 3.79 | 1.73 | 0.08 |
| Imagery*Mismatch vs. neutral | 1.41 | 3.77 | 0.37 | .071 |

**B5. Pleasantness**

B5a. *Model1.lmer = lmer(VasScore ~ Length + (1|Subject) + (1|OdorName), Data, REML=FALSE)*

Random effects Fixed effects

| **Group** | **Var** | ***SD*** |
| --- | --- | --- |
| Subject | 33.2 | 5.76 |
| Odor | 102.3 | 10.11 |

| **Variable** | **Coeff. Est.** | ***SE*** | ***t*** | ***p*** |
| --- | --- | --- | --- | --- |
| Length | 0.03 | 0.35 | 0.08 | 0.94 |

B5b. *Model2.lmer = lmer(VasScore ~ condition + Length + (1|Subject) + (1|OdorName), Data, REML=FALSE)*

Random effects

| **Group** | **Var** | ***SD*** |
| --- | --- | --- |
| Subject | 33.45 | 5.78 |
| Odor | 102.33 | 10.12 |

| **Variable** | **Coeff. Est.** | ***SE*** | ***t*** | ***p*** |
| --- | --- | --- | --- | --- |
| Length | 0.25 | 0.37 | 0.66 | 0.51 |
| Condition: |  |  |  |  |
| Match vs. near-match | 3.26 | 2.16 | 1.51 | 0.13 |
| Match vs. mismatch | 6.86 | 2.03 | 3.37 | < .001 |
| Match vs. neutral | 7.57 | 2.06 | 3.67 | < .001 |
| Near-match vs. mismatch | 3.59 | 2.03 | 1.77 | 0.08 |
| Near-match vs. neutral | 4.30 | 2.01 | 2.14 | 0.03 |
| Mismatch vs. neutral | 0.71 | 1.99 | 0.36 | 0.72 |

Fixed effects

B5c. *Model3.lmer = lmer(VasScore ~ ImageryScore + Length + (1|Subject) + (1|OdorName), Data, REML=FALSE)*

Random effects

| **Group** | **Var** | ***SD*** |
| --- | --- | --- |
| Subject | 27.61 | 5.26 |
| Odor | 102.23 | 10.11 |

Fixed effects

| **Variable** | **Coeff. Est.** | ***SE*** | ***t*** | ***p*** |
| --- | --- | --- | --- | --- |
| Imagery | 5.36 | 2.24 | 2.40 | 0.02 |
| Length | 0.04 | 0.35 | 0.11 | 0.91 |

B5d. *Model4.lmer = lmer(VasScore~ condition + ImageryScore + Length +(1|Subject) + (1|OdorName), Data, REML=FALSE)*

Random effects

| **Group** | **Var** | ***SD*** |
| --- | --- | --- |
| Subject | 27.94 | 5.59 |
| Odor | 102.26 | 10.11 |

Fixed effects

| **Variable** | **Coeff. Est.** | ***SE*** | ***t*** | ***p*** |
| --- | --- | --- | --- | --- |
| Length | 0.23 | 0.37 | 0.63 | 0.53 |
| Imagery | 5.32 | 2.23 | 2.38 | 0.02 |
| Condition: |  |  |  |  |
| Match vs. near-match | 3.23 | 2.16 | 1.5 | 0.13 |
| Match vs. mismatch | 6.84 | 2.03 | 3.37 | < .001 |
| Match vs. neutral | 7.55 | 2.06 | 3.67 | < .001 |
| Near-match vs. mismatch | 3.61 | 2.03 | 1.78 | 0.08 |
| Near-match vs. neutral | 4.31 | 2.02 | 2.15 | 0.03 |
| Mismatch vs. neutral | 0.71 | 1.99 | 0.36 | 0.72 |

B5e. *Model5.lmer = lmer(VasScore ~ condition*ImageryScore + (1|Subject) + (1|OdorName), Data, REML=FALSE)*

Random effects

| **Group** | **Var** | ***SD*** |
| --- | --- | --- |
| Subject | 28.03 | 5.29 |
| Odor | 103.23 | 21.75 |

Fixed effects

| **Variable** | **Coeff. Est.** | ***SE*** | ***t*** | ***p*** |
| --- | --- | --- | --- | --- |
| Length | 0.24 | 0.37 | 0.65 | 0.52 |
| Imagery | 7.48 | 3.58 | 2.09 | 0.04 |
| Condition: |  |  |  |  |
| Match v. near-match | 1.38 | 12.40 | 0.11 | 0.91 |
| Match vs. mismatch | 11.01 | 12.41 | 0.89 | 0.38 |
| Match vs. neutral | 4.23 | 12.37 | 0.34 | 0.73 |
| Near-match vs. mismatch | 11.98 | 12.35 | 0.97 | 0.33 |
| Near-match vs. neutral | 3.11 | 12.40 | 0.25 | 0.80 |
| Mismatch vs. neutral | 15.09 | 12.35 | 1.22 | 0.22 |
| Condition*Imagery: |  |  |  |  |
| Imagery*Match vs. near-match | 0.70 | 4.57 | 0.15 | 0.88 |
| Imagery*Match vs. mismatch | 6.69 | 4.58 | 1.46 | 0.14 |
| Imagery*Match vs. neutral | 1.25 | 4.57 | 0.27 | 0.78 |
| Imagery*Near-match vs. mismatch | 5.94 | 4.57 | 1.30 | 0.19 |
| Imagery*Near-match vs. neutral | 0.52 | 4.59 | 0.11 | 0.91 |
| Imagery*Mismatch vs. neutral | 5.42 | 4.57 | 1.19 | 0.24 |

**B6. Familiarity**

B6a. *Model1.lmer = lmer(VasScore ~ Length + (1|Subject) + (1|OdorName), Data, REML=FALSE)*

Random effects

| **Group** | **Var** | ***SD*** |
| --- | --- | --- |
| Subject | 103.67 | 10.18 |
| Odor | 25.13 | 5.01 |

Fixed effects

| **Variable** | **Coeff. Est.** | ***SE*** | ***t*** | ***p*** |
| --- | --- | --- | --- | --- |
| Length | 0.59 | 0.43 | 1.36 | 0.18 |

B6b. *Model2.lmer = lmer(VasScore ~ condition + Length + (1|Subject) + (1|OdorName), Data, REML=FALSE)*

Random effects

| **Group** | **Var** | ***SD*** |
| --- | --- | --- |
| Subject | 103.57 | 10.18 |
| Odor | 24.92 | 4.99 |

| **Variable** | **Coeff. Est.** | ***SE*** | ***t*** | ***p*** |
| --- | --- | --- | --- | --- |
| Length | 0.70 | 0.47 | 1.50 | 0.13 |
| Condition: |  |  |  |  |
| Match vs. near-match | 1.22 | 2.75 | 0.44 | 0.66 |
| Match vs. mismatch | 4.11 | 2.60 | 1.57 | 0.11 |
| Match vs. neutral | 4.35 | 2.63 | 1.65 | 0.09 |
| Near-match vs. mismatch | 2.89 | 2.60 | 1.11 | 0.27 |
| Near-match vs. neutral | 3.13 | 2.57 | 1.22 | 0.22 |
| Mismatch vs. neutral | 0.24 | 2.55 | 0.09 | 0.93 |

Fixed effects

B6c. *Model3.lmer = lmer(VasScore ~ ImageryScore + Length + (1|Subject) + (1|OdorName), Data, REML=FALSE)*

Random effects

| **Group** | **Var** | ***SD*** |
| --- | --- | --- |
| Subject | 101.44 | 10.07 |
| Odor | 25.09 | 27.91 |

Fixed effects

| **Variable** | **Coeff. Est.** | ***SE*** | ***t*** | ***p*** |
| --- | --- | --- | --- | --- |
| Imagery | 3.37 | 3.60 | 0.94 | 0.35 |
| Length | 0.60 | 0.43 | 1.37 | 0.17 |

B6d. *Model4.lmer = lmer(VasScore~ condition + ImageryScore + Length +(1|Subject) + (1|OdorName), Data, REML=FALSE)*

Random effects

| **Group** | **Var** | ***SD*** |
| --- | --- | --- |
| Subject | 101.33 | 10.07 |
| Odor | 24.88 | 4.99 |

Fixed effects

| **Variable** | **Coeff. Est.** | ***SE*** | ***t*** | ***p*** |
| --- | --- | --- | --- | --- |
| Length | 0.71 | 0.47 | 1.52 | 0.13 |
| Imagery | 3.39 | 3.60 | 0.94 | 0.35 |
| Condition: |  |  |  |  |
| Match vs. near-match | 1.24 | 2.75 | 0.45 | 0.65 |
| Match vs. mismatch | 4.12 | 2.60 | 1.59 | 0.11 |
| Match vs. neutral | 4.36 | 2.63 | 1.66 | 0.10 |
| Near-match vs. mismatch | 2.88 | 2.60 | 1.11 | 0.27 |
| Near-match vs. neutral | 3.12 | 2.57 | 1.21 | 0.23 |
| Mismatch vs. neutral | 0.24 | 2.55 | 0.09 | 0.93 |

B6e. *Model5.lmer = lmer(VasScore ~ condition*ImageryScore + Length + (1|Subject) + (1|OdorName), Data, REML=FALSE)*

Random effects

| **Group** | **Var** | ***SD*** |
| --- | --- | --- |
| Subject | 101.51 | 10.08 |
| Odor | 23.42 | 4.84 |

Fixed effects

| **Variable** | **Coeff. Est.** | ***SE*** | ***t*** | ***p*** |
| --- | --- | --- | --- | --- |
| Length | 0.71 | 0.47 | 1.52 | 0.13 |
| Imagery | 2.40 | 5.07 | 0.47 | 0.64 |
| Condition: |  |  |  |  |
| Match v. near-match | 8.26 | 15.79 | 0.52 | 0.60 |
| Match vs. mismatch | 9.02 | 15.80 | 0.57 | 0.57 |
| Match vs. neutral | 18.13 | 15.76 | 1.15 | 0.25 |
| Near-match vs. mismatch | 0.76 | 15.75 | 0.05 | 0.96 |
| Near-match vs. neutral | 26.38 | 15.78 | 1.67 | 0.09 |
| Mismatch vs. neutral | 27.14 | 15.74 | 1.73 | 0.08 |
| Condition*Imagery: |  |  |  |  |
| Imagery*Match vs. near-match | 2.63 | 5.82 | 0.45 | 0.65 |
| Imagery*Match vs. mismatch | 1.83 | 5.83 | 0.31 | 0.75 |
| Imagery*Match vs. neutral | 8.42 | 5.82 | 1.45 | 0.15 |
| Imagery*Near-match vs. mismatch | 0.80 | 5.82 | 0.14 | 0.89 |
| Imagery*Near-match vs. neutral | 11.05 | 5.83 | 1.90 | 0.06 |
| Imagery*Mismatch vs. neutral | 10.26 | 2.81 | 1.76 | 0.08 |

1. Coefficient could not be estimated due to very large eigenvalues [↑](#footnote-ref-1)
